# Portuguese Man-of-War (*Physalia physalis*) in the Mediterranean: A permanent invasion or a casual appearance?

**DOI:** 10.1038/srep11545

**Published:** 2015-06-25

**Authors:** L. Prieto, D. Macías, A. Peliz, J. Ruiz

**Affiliations:** 1 Instituto de Ciencias Marinas de Andalucía (ICMAN-CSIC); Republica Saharaui 2; Puerto Real (Cádiz) 11519, Spain; 2 European Commission, Joint Research Centre, Institute for Environment and Sustainability, Water Resources Unit, Via E. Fermi 2749, Ispra 21027, Italy; 3 Instituto Dom Luiz, Faculdade de Ciências da Universidade de Lisboa, Campo Grande, Lisboa 1749-016, Portugal

## Abstract

In 2010, the Mediterranean basin experienced Portuguese Man-of-War (*Physalia physalis*) swarms that had dramatic consequences, including the region’s first recorded human fatality attributed to a jellyfish sting. Despite the impact of jellyfish on coastal economic activity and the importance of the tourism industry for the Mediterranean region (accounting for 15% of global tourism), no scientific consensus has been achieved regarding the causes of this episode. Here, we analyse the meteorological and oceanographic conditions of the North-East Atlantic Ocean during the months previous to the appearance of *P. physalis* in the Mediterranean. We simulate the probable drift of Atlantic populations into the Mediterranean basin with a numerical model and compare model results with available observations. We conclude that the summer 2010 *P. Physalis* swarm was the result of an unusual combination of meteorological and oceanographic conditions during the previous winter and not a permanent invasion favoured by climatic changes.

While swimming in the waters off the Italian island of Sardinia in August 2010, a woman suffered an allergic reaction and died after being stung by a Portuguese Man-of-War (*Physalia physalis*)^1^. *P. physalis*, a pleustonic colony of polypoid and medusoid organisms, is equipped with a particularly potent toxin that is potentially deadly to humans^2^,^3^ (more details in Supplementary Information).

In addition to this isolated fatality, an unusual number of *P. physalis* sightings were also recorded along the coast of the Mediterranean Sea, Iberian Peninsula (both along the Atlantic and Mediterranean coast lines) and Canary Islands (Fig. 1a, Supplementary Table 1) in the year 2010. *P. physalis* is not native to the Mediterranean; it is usually found in the tropical and subtropical areas of the Pacific, Atlantic and Indian Oceans^4^, ranging from 55 °N to 40 °S. Hence, tourism, a major economic sector in Europe with an annual flow of tourists from northern to southern Europe (Mediterranean coastal countries) that accounts for one in every six tourist arrivals worldwide^5^, could be under a potential threat by this foreign species^6^.

To elucidate the likelihood of this hypothesis, we performed a comprehensive analysis of the environmental conditions (biotic and abiotic) associated with the *P. physalis* swarm events in 2010. We first compiled all of the available information regarding *P. physalis* arrivals along the coast over several years. Second, we examined the specific 2010 climatic/oceanographic conditions within a wider temporal context. Finally, we performed a model simulation of the drifting of individual siphonophores coupling an hydrodynamic model to an individual based model. The hydrodynamic model simulates water movement and physical oceanic conditions of the Gulf of Cadiz and Alboran Sea regions while the individual based model track the drifting path of each individual colony under the combined action of currents and wind drift. Model estimated beaching patterns of *P. physalis* were compared with available observations at both sides of the Strait of Gibraltar for winter/spring of year 2010.

## Results

A plausible explanation for the occurrence of the Portuguese Man-of-War within the Mediterranean Basin in summer 2010 is that specific climatic and oceanographic conditions during the previous winter in the North Atlantic favoured the transport of this jellyfish organism into the Mediterranean. The North Atlantic Oscillation index (NAO) is one of the major modes of variability in the Northern Hemisphere atmosphere^7^ and is particularly significant in winter (December to March) when it exerts a strong control on the climate of Western Europe by regulating the intensity of zonal winds and precipitation patterns^8^. The 2009–2010 winter had one of the most negative NAO indices (−4.64) measured during the nearly 150-year record^9^ (Supplementary Fig. 1). This climatic condition favoured a stormy mid-latitude Atlantic, with increased storm activity and rainfall in southern Europe, the western Mediterranean and North Africa^10^.

**Figure 1:**
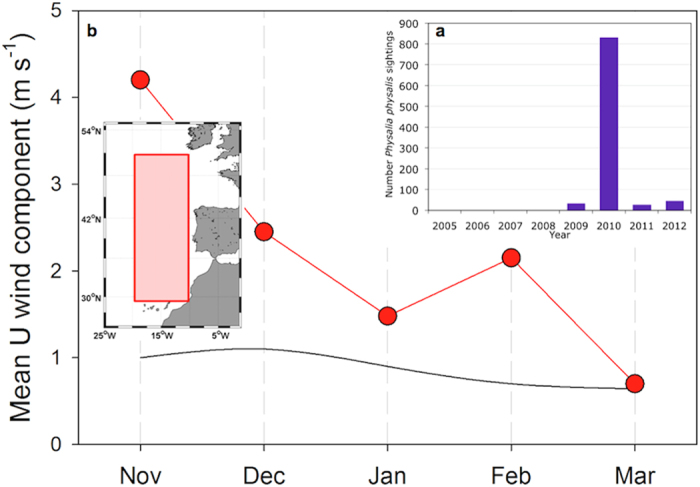
Wind and Occurrence of Portuguese Man-of-War. (**a**) Total number of *Physalia physalis* sightings on the coast of the Mediterranean Sea, Iberian Peninsula (both Atlantic and Mediterranean coastlines) and Canary Islands during eight consecutive years (2005–2012). The year 2010 is an anomalous year in terms of the frequency of occurrences and in the total number of colonies arriving (more than 100,000 colonies) compared to 2009 and 2011, which featured less than 60 colonies. (**b**) The black line is the monthly climatology from 1979 to 2012 for the wind (U component) in the Atlantic Ocean from 50 °N to 28 °N and from −20 °W to −10 °W (red square in the map). The red line shows the data from the 2009–2010 winter when westerlies (positive values) were much stronger in the entire basin compared to the average. The data are ERA-Interim daily analysis products (http://www.ecmwf.int/en/research/climate-reanalysis/era-interim) downloaded on 12 June, 2013. The map in the inset was created by the authors using the *m-map* toolbox included in Matlab®.

Wind data from the ERA-interim analysis provided by the European Center for Medium-range Weather Forecast (ECMWF) for the North-East Atlantic (29 °N–51 °N, 20 °W–10 °W) illustrate that the 2009–2010 winter indeed featured an anomalous intensity of westerly winds (Fig. 1b), with mean values between 1.5 and 4 times higher than the long-term (1979–2012) average.

To specify the moment to begin the model simulation, we analysed the wind patterns in the NE Atlantic area (29 °N–51 °N, 20 °W-10 °W) (Fig. 1, red square in inset). In addition to the previously mentioned anomalous intensity of westerly winds between November and December 2009, during the first 25 days of January 2010 the daily mean kinematic wind stress in the southwestern corner of Portugal (at −10 °W and between 37 °N and 38 °N) indicated continuously blowing westerlies with no changes in direction (supplementary Fig. 2). Therefore, we decided that these continuous westerly winds could have likely moved the open-ocean *P. physalis* population towards the mainland. Consequently, the first day of the simulation was 26 January 2010.

To determine the initial position of the colony along the Portuguese coast we used the singular arrival of 10,373 *P. physalis* colonies to the continuously monitored shoreline of Doñana National Park (SW Iberian Peninsula) during 22–26 February, 2010. A backward analysis of simulated colonies’ drift determined that this population was most likely located off the southwestern Portuguese coast one month earlier (see Methods for details).

**Figure 2:**
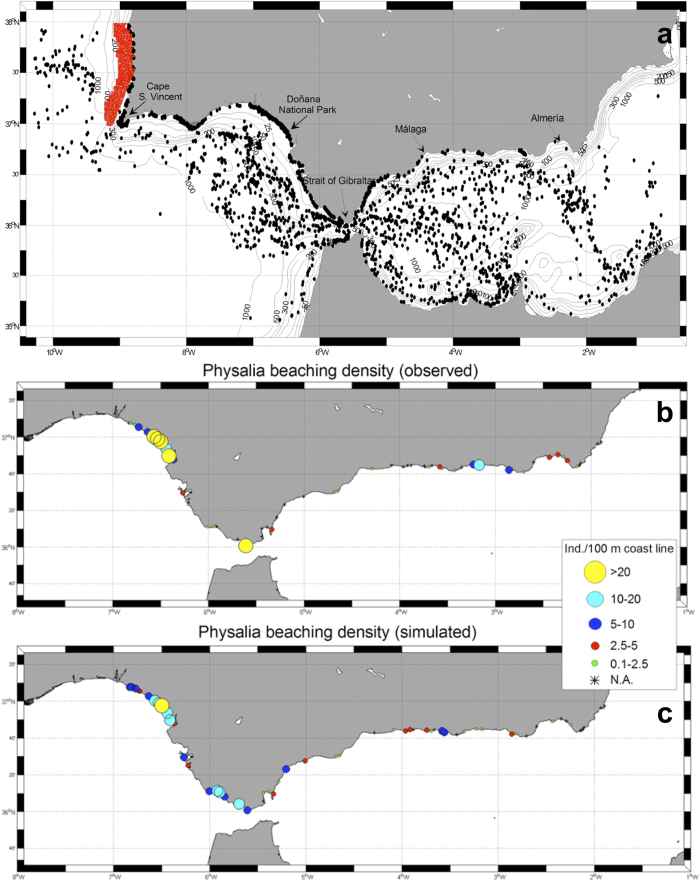
Simulation of Portuguese Man-of-War jellyfish drifting and actual beachings during January-March 2010. (**a**) The virtual position of *P. physalis* on 26 January 2010 (beginning of the simulation) in small red dots and on 30 March, 2010 (end of the simulation) in black dots. The model combines the effect of hydrodynamics with the effect of wind in moving each colony from the Atlantic to the Mediterranean through the Strait of Gibraltar. The cumulative density (colonies per 100 m of coastline) of the observed (**b**) and simulated (**c**) *P. physalis* arrivals to the Atlantic and Mediterranean coasts of the South Iberian Peninsula are shown. Observed and simulated abundance of *P. physalis* arrivals are aggregated at beach level (beaches identified in Supplementary table 1). The arrivals of *P. physalis* occurred from west to east both in the observations and in the simulation (see Supplementary Figure 3) between 22 February and 30 March. The simulation is the result of an IBM coupled to Regional Oceanographic Model that includes the effect of the wind and currents (more details in Methods). Maps were created by the authors using the *m-map* toolbox included in Matlab®.

We then initialised the model run by seeding that particular region of the southwestern Portugal with a *P. physalis* population of 25,000 colonies extending from the coast (20 m depth) to the continental slope (200 m depth) at the end of January 2010, as discussed above (red circles in Fig. 2a). We projected this simulation forward and ran it until the end of March 2010 (black circles in Fig. 2a).

The simulated *P. physalis* beaching pattern is very similar to the observations along the coast (Fig. 2b,c and Supplementary Fig. 3). Some of the discrepancies in the densities are very likely due to sampling deficiencies; certain beaches were sampled only once, and several tidal cycles occurred between the predicted date of beaching by the numerical model and the actual sampling date. This result suggests that some of the stranded *P. physalis* colonies could have been washed out again by tidal movement, decreasing the observed density.

**Figure 3:**
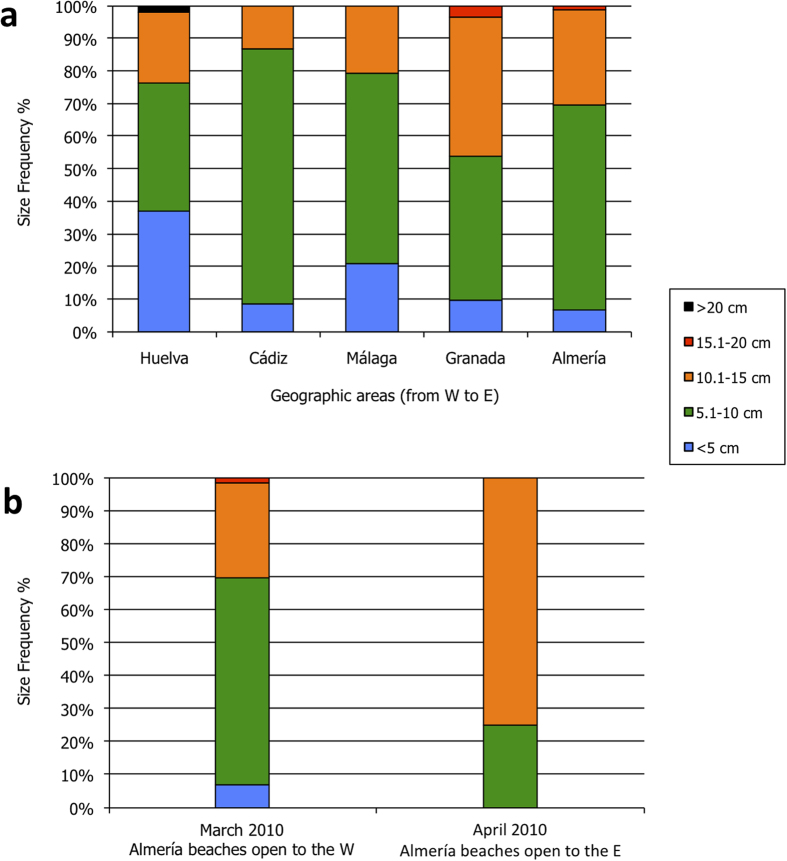
Size frequency of the stranded Portuguese Man-of-War. (**a**) Size frequency of all of the colonies of *P. physalis* stranded during February-March 2010 in the Iberian Peninsula, as grouped by province from west to east. (**b**) Comparison of the size frequency of *P. physalis* stranded in the easternmost province in March and April 2010. Overall, the size frequency of the population stranded shifted toward larger colonies.

Additionally, a few colonies again arrived at the coast a month later (April 2010) in the easternmost end of the Alboran Sea and with a larger mean size than the ones previously detected (Fig. 3).

## Discussion

This simulation and our analysis of the meteorological conditions during this particular year seem to indicate that the main mechanism involved in the massive arrival of *P. physalis* to the coast was the zonal wind that pushed populations from the open ocean toward the Iberian Margin. In line with this hypothesis, *P. physalis* arrivals to the southern Iberian coast, on both the Atlantic and Mediterranean (in the Alboran Sea) sides, were registered during February and March of 2010 (Supplementary Table 1), a period with strong westerly events in the region, as indicated above (Fig. 1b).

However, we cannot exclude the role of the open-ocean currents during that particular year, which may also have been strong. The relationship between NAO and open ocean circulation in the North-East Atlantic is nonetheless less straightforward^11^. The core of the eastern side of the Azores Current fluctuates in latitude, with fluctuations in its axis of a few degrees from year to year^12^. However, the role climatic oscillations such as NAO play in these fluctuations is not yet well established.

Due to their enlarged pneumatophores (the sail-shaped structure filled with gas^13^) *P. physalis* individuals are advected under the direct influence of wind drag, particularly by moderately strong winds (i.e., approximately 5 m/s)^13^, that generates drifting velocities that are well above typical ocean currents speed. As soon as the individuals approach the coast, they begin to feel the effect of the slope-shelf currents, which are strong (even under weak winds) and can compete with the wind with regard to the dispersal of the jellyfish, primarily due to the colonies’ long filaments. We have not considered the two possible configurations of the colonies (*right* and *left*^13^) that make them sail at a certain angle to the wind direction (10–15°) because this angle decreases with intense winds^14^ (such as those during storm events) and because of the shorter distances travelled by the colonies under the main influence of winds from the shelf to the coast (see Methods for a more detailed description).

The main upper slope current along the Southwestern Iberian Margin and Gulf of Cadiz (the Gulf of Cadiz Slope Current, GCC) is linked to the inflow into the Mediterranean^15^,^16^. Indeed, a larger inflow will induce a stronger GCC, which will generate a suction mechanism from a larger area and from larger distances (poleward along the Southwestern Iberian Margin) from the Strait of Gibraltar. Within this context, if a large population is ‘available’ along the Southwestern Iberian Margin, a strong inflow (in this case enhanced by persistent zonal winds) will produce a massive advection into the Mediterranean.

Therefore, our analysis and simulations clearly support that the exceptional occurrence of the Portuguese Man-of-War within the Mediterranean in summer 2010 could be explained by the unique climatic conditions during the previous winter. Currents and winds acted together to push Atlantic colonies through the Strait of Gibraltar and into the Mediterranean basin. This is further supported by the larger size class of the jellyfish reaching the coasts of the Alboran Sea in April 2010 (Fig. 3), almost two months later than the initial beaching event. This indicates that they were likely part of the same population that entered the Mediterranean from the Atlantic and that they had spent several weeks trapped in the anticyclonic circulation of the Alboran Sea Gyres before reaching the coast. This second influx of colonies appeared along the beaches open to the east (Fig. 3b), and the colonies were pushed to shore by persistent winds with an easterly component.

The stranding pattern of *P. physalis* the next year (2011) inside the Mediterranean comprised only eight sightings, and a total of 17 colonies (Supplementary Table 1). Only two colonies were observed inside the Mediterranean during 2012. These figures are similar to the strandings in 2009 (eight sightings totalling 57 colonies), indicating that the colony density did not permanently increase inside the basin after 2010. Moreover, only one *P. physalis* colony was observed in the Mediterranean Basin east of the Balearic Sea in 2011 and zero in 2012^17^,^18^. We therefore propose that the presence of *P. physalis* along Mediterranean beaches will not constitute a continuous problem.

Nevertheless, the possibility that the particular/unique conditions that occurred during 2010 (and permitted this intrusion) will become more frequent greatly depends on the projected NAO patterns in future climate scenarios. In this sense, a few studies have shown increasingly positive trends in the NAO index in simulations with increased greenhouse gas emissions, though this is not true in all models, and the magnitude and character of the changes vary across the models^19^. Indeed, NAO projections remain one of the key uncertainties in future climate projections^20^. Therefore, unless the NAO drifts toward more negative values under the influence of climate change recreating these so far unique meteorological conditions increasingly frequently, the 2010 *P. physalis* swarm event is unlikely to re-occur on a regular basis.

## Methods

*P. physalis* sightings were compiled for eight years from different sources: media, national and regional agencies and personal communication. The unique event of February-April 2010 was carefully monitored by the Technicians of the Consejeria de Medio Ambiente from the regional government of Andalucía, which monitored the entire coast and counted and measured all stranded colonies. Additionally, *P. physalis* sightings were analysed from the database of the Jellywatch Program^17^ (http://jellywatch.org/) for the Mediterranean basin.

The wind data were obtained from the ERA-interim reanalysis provided by the European Center for Medium-range Weather Forecast (ECMWF) for the North-East Atlantic (29 °N–51 °N, 20 °W–10 °W) (freely available at http://apps.ecmwf.int/datasets/, data downloaded on 12 June, 2013)

The ocean simulations were conducted using a hydrodynamic model^15^ consisting of an ROMS^21^-based numerical simulation with 2-km resolution forced with realistic winds (ASCAT) and heat fluxes from ERA-interim^22^.

Individual-Based Model (IBM) simulations were performed using the free modelling tool *Ichthyop* v.3.1.^23^ (available at http://www.brest.ird.fr/ressources/ichthyop) coupled off-line with the ocean model described above. We simulated each *P. physalis* colony as a virtual individual floating at the sea surface and being advected by the joint effects of the surface currents (computed by the ROMS model) and wind drag (from the ASCAT database). To estimate the drag effect of the wind, we assumed that each colony was transported in the wind direction at 10% of the wind velocity^13^. *P. physalis* individuals have been described to present the pneumatophore (the ‘sail’) with a deviation (to the left or to the right) with respect to the tentacles that drives them at a certain angle (10–15°) to the wind direction^13^. We have not included this characteristic in our IBM because the simulation started with the population already in the vicinity of the coast, where current dragging is the primary mechanism that determines an individual’s path, as described above. The wind effect is mostly relevant during the intense winds that pushed the colonies towards the beach along a relatively short distance. Under these strong wind conditions, the relative angle between the sail and the wind decreases^14^ and the deviation effect described above should only marginally affect the exact beaching location of the individuals. Indeed, sensitivity experiments assuming different percentages of left and right individuals show no significant alteration in the beaching pattern (results not shown).

The virtual individuals were not allowed to grow or die but were merely inert surface drifters. This is a reasonable assumption given the short duration (~ 2 months) of the simulation. When a virtual colony reached the model land boundary, the time and coordinates were recorded; these stranding data were then compared to the observations in the field.

## Additional Information

**How to cite this article**: Prieto, L. *et al.* Portuguese Man-of-War (*Physalia physalis*) in the Mediterranean: A permanent invasion or a casual appearance? *Sci. Rep.* **5**, 11545; doi: 10.1038/srep11545 (2015).

## Supplementary Material

Supplementary Information

## References

[b1] Haywood L. Killer ‘jellyfish’ strikes in Italy, http://www.thesun.co.uk/sol/homepage/news/3112641/Killer-jellyfish-strikes-in-Italy.html (2010). Accessed on 05/03/2011.

[b2] Burnett J. Medical aspects of jellyfish envenomation: pathogenesis, case reporting and therapy. Hydrobiology 155, 1–9 (2000).

[b3] Edwards L. & Hessinger D. A. Portuguese Man-of-war (*Physalia physalis*) venom induces calcium influx into cells by permeabilizing plasma membranas. Toxicon 38, 1015–1028 (2000). 10708794 10.1016/s0041-0101(99)00213-5

[b4] Kirkpatrick P. A. & Pugh P. R. Siphonophores and Velellids. Synopses of the British Fauna New Series 29, 1–154 (1984).

[b5] Ciscar J.-C. *et al.* Physical and economic consequences of climate change in Europe. Proc. Natl Acad. Sci. USA 108, 2678–2683 (2011). 21282624 10.1073/pnas.1011612108 PMC3041092

[b6] Schrope M. Attack of the blobs. Nature 482, 20–21 (2012). 22297950 10.1038/482020a

[b7] Walker G. T. & Bliss E. W. World weather V. Mem. Royal Meteorological Society 45, 53–84 (1932).

[b8] Barnston A. G. & Livezey R. E. Classification, seasonality and persistence of low-frequency atmospheric circulation patterns. Mon. Weather. Rev. 115, 1083–1126 (1987).

[b9] Hurrell J. & National Center for Atmospheric Research Staff (Eds). The Climate Data Guide: Hurrell North Atlantic Oscillation (NAO) Index (station-based), https://climatedataguide.ucar.edu/guidance/hurrell-north-atlantic-oscillation-nao-index-station-based (2012). Accessed on 21/03/2013.

[b10] Stenseth N. C. *et al.* Studying climate effects on ecology through the use of climate indices: the North Altlantic Oscillation, El Niño Southern Oscillation and beyond. Proc. R. Soc. B-Biol. Sci. 270, 2087–2096 (2003). 10.1098/rspb.2003.2415 PMC1691494 14561270

[b11] Volkov D. L. Interannual variability of the altimetry-derived eddy field and surface circulation in the Extratropical North Atlantic Ocean in 1993–2001, J. Phys. Oceanogr. 35, 405–426 (2005).

[b12] Barbosa Aguiar A. C., Peliz A., Cordeiro Pires A. & Le Cann B. Zonal structure of the mean flow and eddies in the Azores Current system. J. Geophys. Res. 116, C02012, 10.1029/2010JC006538 (2011).

[b13] Iosilevskii G. & Weihs D. Hydrodynamics of sailing of the Portuguese man-of-war *Physalia physalis*. J. R. Soc. Interface 6, 613–626 (2009). 19091687 10.1098/rsif.2008.0457 PMC2696138

[b14] Totton A. K. & Mackie G. O. Studies on Physalia physalis. Discovery reports 30, pp. 301–408. Cambridge, UK: Cambridge University Press (1960).

[b15] Peliz A., Boutov D., Cardoso R., Delgado J. & Soares P. M. M. The Gulf of Cadiz-Alboran Sea sub-basin: Model setup, exchange and seasonal variability. Ocean Model. 61, 49–67; 10.1016/j.ocemod.2012.10.007 (2013).

[b16] Peliz A. *et al.* Surface circulation in the Gulf of Cadiz. Part 2: Inflow/outflow coupling and the Gulf of Cadiz Slope Current. J. Geophys. Res. 114, C03011; 10.1029/2008JC004771 (2009).

[b17] Jellywatch Program, http://jellywatch.org/, (2012). Accessed on 12/09/2012.

[b18] Rare Portuguese man o’ war jellyfish sighted off Marsamxett, http://www.timesofmalta.com/articles/view/20110309/local/rare-portuguese-man-o-war-jellyfish-sighted-off-marsamxett.353899 (2011). Accessed on 17/10/2011.

[b19] IPCC (2001) Climate Change 2001: The scientific Basis. Contribution of Working Group I to the Third Assessment Report of the Intergovernmental Panel on Climate Change (eds Core Writing Team Houghton, J.T. *et al.*) (IPCC, NY, 2001).

[b20] IPCC (2007) Climate Change 2007: Synthesis Report. Contribution of Working Groups I, II and III to the Fourth Assessment Report of the Intergovernmental Panel on Climate Change (eds Core Writing Team Pachauri, R.K. & Reisinger, A.) (IPCC, NY, 2007).

[b21] Shchepetkin A. F. & McWilliams J. C. The regional oceanic modeling system (roms): a split-explicit, free-surface, topography-following-coordinate oceanic model. Ocean Model. 9, 347–404 (2005).

[b22] Dee D. P. *et al.* The ERA-Interim reanalysis: configuration and performance of the data assimilation system. Q. J. R. Meteorol. Soc. 137, 553–597; 10.1002/qj.828 (2011).

[b23] Lett C. *et al.* A lagrangian tool for modelling ichthyoplankton dynamics. Environ. Modell. Softw. 23, 1210–1214 (2008).

