# Supplementary material for: Portuguese Man-of-War (Physalia physalis) in the Mediterranean: A permanent invasion or a casual appearance?
Source: Sci Rep. 2015 Jun 25;5:11545. doi: 10.1038/srep11545 (PMC4480229; doi:10.1038/srep11545)
Supplement: Supplementary Information [file srep11545-s1.pdf]

Portuguese Man-of-War (*Physalia physalis*) in the Mediterranean: A permanent invasion or a casual appearance?

L. Prieto, D. Macías, A. Peliz and J. Ruiz

SUPPORTING INFORMATION

The Portuguese Man-of-War toxin: description and envenomation syndromes.

The Portuguese Man-of-War, *Physalia physalis*, possesses a singularly potent toxin (Brunett 2000; Edwards & Hessinger 2000). The major protein of the venom is physalitoxin, a potent hemolysin (Tamkun & Hessinger 1981), contained in the nematocysts. The chemical characterization (Stillway 1974), collagenase activity[28] and cloning and functional expression of the cnidocytes (Bouchard *et al*. 2006) have been described. The nematocyst venom of *P. physalis* is lethal to animals and humans (Edwards & Hessinger 2000), and the envenomation syndromes in humans are extensive (Burnett 2000). Fatal reactions include immediate toxin-induced cardiac arrest and delayed renal failure. Systemic reactions include toxin-induced Irukandji reaction and respiratory acidosis, and local reactions include recurrent toxin-induced skin, mucosa and cornea reactions of up to four episodes, distant site reactions and local lymphadenopathy. Chronic reactions include contractions, vascular spasm and mononeuritis, post-episode dermatitis and granuloma annulare. Urticaria occurs as a reaction to ingestion.

References used in Supplementary material and not in the paper:

1. Tamkun, M.M. & Hessinger, D.A. (1981) Isolation and partial characterization of a hemolytic and toxic protein from the nematocyst venom of the Portuguese Man-of-war, *Physalia physalis*. *Biochim. Biophys. Acta,* 667, 87-98 .

2. Stillway, L.W. (1974). Nematocyst lipids of the Portuguese Man-of-war *Physalia physalis**. *Comp. Biochem. Physiol.*, 48B, 35-38 .

3. Lal, D.M., Calton, G.J., Neeman, I. & Burnett, J.W. (1981). Characterization of *Physalia physalis* (Portuguese Man-O'War) nematocyst venom collagenase. *Comp. Biochem. Physiol.*, 70B, 635-638 .

4. Bouchard. C., Price, R.B., Moneypenny, C.G., Thompson, L.F., Zillhardt, M., Stalheim, L. *et al*. (2006). Cloning and functional expression of voltage-gated ion channel subunits from cnidocytes of the Portuguese Man O'War *Physalia physalis*. *J. Exp. Biol.*, 209, 2979-2989 .

Supplementary Table1 | List of sightings of Portuguese Man-of-War. Total number of *Physalia physalis* sightings along the coast of the Mediterranean Sea, Iberian Peninsula (both at the Atlantic and Mediterranean coastlines) and Canary Islands. The quantity references are as follows: 0 (zero colonies), 1 (1 colony), 2 (2-5 colonies), 3 (6-10 colonies), 4 (11-99 colonies) and 5 (>100 colonies). Note: 559 jellyfish sightings were observed from 2005 to 2007, but none of them correspond to *Physalia physalis*. Nan: not specified. MMA: Spanish Ministry of Environment.

Day	Month	Year	Quant	Latitude	Longitude	Beach/Port	Province	Reference
23	8	2008	1	36.7316	-3.7454	La Herradura	Granada	MMA
23	8	2008	3	36.6884	-3.4682	Salobreña	Granada	MMA
26	8	2008	Nan	36.5870	-4.5257	Benalmádena	Málaga	MMA
26	8	2008	1	36.7290	-3.6553	Torrenueva	Granada	MMA
29	8	2008	1	39.3654	-9.3534	Peniche	Portugal	http://laurindaalves.blogs.sapo.pt/121964.html
5	2	2009	3	36.4421	-6.2382	Camposoto	Cádiz	pers comm
26	4	2009	1	37.6000	-0.7453	Las Cañas	Murcia	http://www.laverdad.es/murcia/20090427/local/region/aparece-nuevo-ejemplar-carabela-200904271709.html
26	4	2009	1	37.8243	-0.7603	San Pedro del Pinatar	Murcia	http://www.laverdad.es/murcia/20090427/local/region/aparece-nuevo-ejemplar-carabela-200904271709.html
27	4	2009	4	37.6099	-0.7251	Coast	Murcia	http://www.abc.es/hemeroteca/historico-27-04-2009/abc/Nacional/avistan-en-la-costa-murciana-unas-medusas-cuya-picadura-puede-ser-mortal-_92478111718.html
3	5	2009	1	37.3999	-1.5806	Aguilas	Murcia	http://www.abc.es/hemeroteca/historico-27-04-2009/abc/Nacional/avistan-en-la-costa-murciana-unas-medusas-cuya-picadura-puede-ser-mortal-_92478111718.html
3	6	2009	NaN	28.1446	-15.4325	Las Canteras	Gran Canaria	MMA
19	6	2009	2	35.8934	-5.3112	Sports Port	Ceuta	MMA
29	6	2009	1	37.8575	-0.7622	Cala del Gato	Murcia	http://www.laverdad.es/murcia/20090630/local/orihuela/carabela-portuguesa-pica-playa-200906301633.html
30	6	2009	2	28.4824	-16.2324	Sports Port	St Cruz Tenerife	MMA
16	7	2009	2	28.5360	-16.3926	La Barranquera	St Cruz Tenerife	MMA
21	7	2009	NaN	27.7664	-15.5499	Las Burra	Gran Canaria	MMA
24	7	2009	3	28.4804	-16.2399	Club Nautico	St Cruz Tenerife	MMA
30	7	2009	2	37.7310	-0.7370	La Manga	Murcia	MMA
30	7	2009	5	39.4330	-9.2409	Foz do Arelho	Portugal	http://www.destak.pt/artigo/37156-caravelas-portuguesas-continuam-a-dar-a-costa
4	8	2009	2	28.5360	-16.3926	La Barranquera	St Cruz Tenerife	MMA
11	8	2009	3	43.5612	-7.1907	Barreiros	Lugo	http://www.lavozdegalicia.es/amarina/2009/08/12/0003_7902617.htm
13	8	2009	2	43.4757	-3.7828	Santander	Cantabria	MMA
24	8	2009	1	43.4710	-3.9313	Portio, Piélagos	Cantabria	MMA
26	8	2009	1	43.3523	-3.1362	Muskiz	Vizcaya	MMA
26	8	2009	2	43.4316	-3.8029	Sports Port	Cantabria	MMA
27	8	2009	2	43.4672	-3.7702	Isla de la Torre	Cantabria	MMA
27	8	2009	1	43.4757	-3.7828	Santander	Cantabria	MMA
29	8	2009	1	43.4922	-3.5391	Joyel, Noja	Cantabria	MMA
31	8	2009	1	43.3869	-3.2248	Castro Urdiales	Cantabria	MMA
1	9	2009	2	28.6394	-17.7571	Breña Baja	St Cruz Tenerife	MMA
1	9	2009	2	28.6394	-17.7571	Breña Baja	St Cruz Tenerife	MMA
5	9	2009	1	43.4461	-3.9716	Valdearenas, Piélagos	Cantabria	MMA
6	9	2009	1	43.4031	-3.3207	Arenillas, Castro Urdiales	Cantabria	MMA
10	9	2009	1	43.3747	-3.2113	Brazomar, Castro Urdiales	Cantabria	MMA
11	9	2009	1	43.4356	-4.0399	La Concha, Suances	Cantabria	MMA
11	9	2009	1	43.4311	-4.0755	Tagle, Suances	Cantabria	MMA
14	9	2009	1	43.3834	-3.0053	La Salvaje, Sopelana	Vizcaya	MMA
15	9	2009	1	43.2878	-2.1707	Zarautz	Guipúzcoa	MMA
22	2	2010	5	36.9907	-6.5499	Matalascañas	Huelva	Consejería de Medio Ambiente
22	2	2010	5	36.9843	-6.5331	Ranchos	Huelva	Consejería de Medio Ambiente
22	2	2010	5	36.9558	-6.4952	Torrecarbonero	Huelva	Consejería de Medio Ambiente
22	2	2010	5	36.8902	-6.4337	Zalabar	Huelva	Consejería de Medio Ambiente
22	2	2010	5	36.8280	-6.4031	Inglesillo	Huelva	Consejería de Medio Ambiente
22	2	2010	0	36.7966	-6.3748	Malandar	Huelva	Consejería de Medio Ambiente
23	2	2010	5	37.0640	-6.6758	Asperillo occidental	Huelva	Consejería de Medio Ambiente
23	2	2010	5	37.0415	-6.6325	Asperillo oriental	Huelva	Consejería de Medio Ambiente
25	2	2010	5	36.8736	-6.4329	Doñana	Huelva	pers communication
26	2	2010	0	37.1347	-6.8374	Mazagón	Huelva	Consejería de Medio Ambiente
26	2	2010	5	37.1049	-6.7587	Rompeculos	Huelva	Consejería de Medio Ambiente
2	3	2010	3	36.1389	-5.3408	Gibraltar	Cadiz	http://www.publico.es/ciencias/298861/alerta/litoral/andaluz/medusa/peligrosa
2	3	2010	0	37.1347	-6.8374	Mazagón	Huelva	Consejería de Medio Ambiente
2	3	2010	3	37.1270	-6.8084	Alcor	Huelva	Consejería de Medio Ambiente
2	3	2010	4	37.1105	-6.7707	Mazagón	Huelva	Consejería de Medio Ambiente
2	3	2010	3	37.0954	-6.7396	Arenosillo	Huelva	Consejería de Medio Ambiente
3	3	2010	2	37.0002	-8.0032	Faro	Portugal	http://www.meteopt.com/forum/seguimento-meteorologico/seguimento-sul-marco-2010-a-4317-3.html
3	3	2010	4	36.1844	-6.0081	Caños de Meca	Cadiz	Consejería de Medio Ambiente
3	3	2010	2	36.1868	-5.9447	Hierbabuena	Cadiz	Consejería de Medio Ambiente
3	3	2010	0	36.1864	-5.9226	Barbate	Cadiz	Consejería de Medio Ambiente
3	3	2010	4	36.1864	-5.9226	Barbate	Cadiz	Consejería de Medio Ambiente
3	3	2010	4	36.1359	-5.8488	Zahara	Cadiz	Consejería de Medio Ambiente
3	3	2010	0	36.0684	-5.6930	Valdevaqueros	Cadiz	Consejería de Medio Ambiente
3	3	2010	0	36.7396	-4.0904	Torre del Mar	Malaga	Consejería de Medio Ambiente
3	3	2010	0	36.7266	-3.9563	Lindes	Malaga	Consejería de Medio Ambiente
3	3	2010	0	36.7377	-3.9262	Calaceite	Malaga	Consejería de Medio Ambiente
3	3	2010	0	36.7435	-3.9009	Playazo	Malaga	Consejería de Medio Ambiente
3	3	2010	0	36.7316	-3.7454	La Herradura	Granada	Consejería de Medio Ambiente
3	3	2010	4	36.7292	-3.6955	Peñón del Santo	Granada	Consejería de Medio Ambiente
3	3	2010	0	36.7383	-3.6687	Velilla	Granada	Consejería de Medio Ambiente
3	3	2010	4	36.7322	-3.5882	Charca	Granada	Consejería de Medio Ambiente
3	3	2010	2	36.7135	-3.5489	Granada	Granada	Consejería de Medio Ambiente
3	3	2010	4	36.7293	-2.8877	Balerma	Almeria	Consejería de Medio Ambiente
3	3	2010	1	36.7045	-2.8066	Almerimar	Almeria	Consejería de Medio Ambiente
3	3	2010	4	36.6831	-2.7005	Punta Entinas	Almeria	Consejería de Medio Ambiente
3	3	2010	0	36.7645	-2.6046	Roquetas	Almeria	Consejería de Medio Ambiente
3	3	2010	4	36.8366	-2.3813	El Alquián	Almeria	Consejería de Medio Ambiente
3	3	2010	2	36.8209	-2.2915	Torregarcía	Almeria	Consejería de Medio Ambiente
3	3	2010	4	36.8173	-2.4371	El Charco	Almeria	Consejería de Medio Ambiente
4	3	2010	0	36.1664	-5.4374	Getares	Cadiz	Consejería de Medio Ambiente
4	3	2010	0	36.1573	-5.4438	Palmones	Cadiz	Consejería de Medio Ambiente
4	3	2010	0	36.1801	-5.3854	Pte. Mayorga	Cadiz	Consejería de Medio Ambiente
4	3	2010	4	36.1775	-5.3357	La Atunara	Cadiz	Consejería de Medio Ambiente
4	3	2010	0	36.2826	-5.2788	Sotogrande	Cadiz	Consejería de Medio Ambiente
4	3	2010	0	36.3817	-5.2105	Torre de la Sal	Malaga	Consejería de Medio Ambiente
4	3	2010	0	36.4663	-4.9957	Guadalmina	Malaga	Consejería de Medio Ambiente
4	3	2010	4	36.5036	-4.6758	Mijas	Malaga	Consejería de Medio Ambiente
4	3	2010	1	36.5083	-4.6372	Calaburras	Malaga	Consejería de Medio Ambiente
4	3	2010	3	36.7128	-4.3122	Rincón	Malaga	Consejería de Medio Ambiente
4	3	2010	2	36.6954	-3.4451	Carchuna	Granada	Consejería de Medio Ambiente
4	3	2010	0	36.7229	-3.3563	Castell de Ferro	Granada	Consejería de Medio Ambiente
4	3	2010	4	36.7497	-3.2391	Melicena	Granada	Consejería de Medio Ambiente
4	3	2010	4	36.7461	-3.1738	La Rábita	Granada	Consejería de Medio Ambiente
4	3	2010	4	36.8259	-2.4500	Zapillo	Almeria	Consejería de Medio Ambiente
4	3	2010	2	36.7286	-2.1547	Monsul	Almeria	Consejería de Medio Ambiente
5	3	2010	3	35.8934	-5.3112	Ceuta	Ceuta	http://www.abc.es/agencias/noticia.asp?noticia=333982
5	3	2010	4	36.4924	-6.2701	Cortadura	Cadiz	Consejería de Medio Ambiente
5	3	2010	2	36.4421	-6.2382	Camposoto	Cadiz	Consejería de Medio Ambiente
5	3	2010	4	36.0209	-5.6159	Los Lances	Cadiz	Consejería de Medio Ambiente
5	3	2010	0	36.7431	-2.1187	Genoveses	Almeria	Consejería de Medio Ambiente
5	3	2010	0	36.7592	-2.1051	San José	Almeria	Consejería de Medio Ambiente
5	3	2010	0	36.9376	-1.9341	Agua Amarga	Almeria	Consejería de Medio Ambiente
5	3	2010	0	36.9863	-1.9015	Punta Muertos	Almeria	Consejería de Medio Ambiente
10	3	2010	3	36.7232	-4.1291	Almayate	Malaga	http://www.elmundo.es/elmundo/2010/03/10/andalucia_malaga/1268241604.html
20	3	2010	1	35.8934	-5.3112	Ceuta	Ceuta	http://www.abc.es/agencias/noticia.asp?noticia=333982
25	3	2010	3	36.1389	-5.3408	Gibraltar	Cadiz	http://www.andaluciainformacion.es/portada/?a=115452&i=17&f=0
25	3	2010	1	36.5500	-2.46	Alborán	Almería	Consejería de Medio Ambiente
26	3	2010	2	36.8315	-2.3173	Retamar	Almería	Consejería de Medio Ambiente
26	3	2010	2	36.8088	-2.4306	Salida Andarax	Almería	Consejería de Medio Ambiente
27	3	2010	2	36.7470	-3.0529	El Lance, Adra	Almería	Consejería de Medio Ambiente
4	4	2010	3	42.2495	-8.7949	Areamilla Cangas	Pontevedra	http://www.farodevigo.es/portada-o-morrazo/2010/04/04/proteccion-civil-retira-47-medusas-muertas-playa-areamilla/425879.html
5	4	2010	3	35.8934	-5.3112	Ceuta	Ceuta	http://www.abc.es/agencias/noticia.asp?noticia=333982
11	4	2010	2	36.7293	-2.8877	Balerma, El Ejido	Almería	Consejería de Medio Ambiente

16	4	2010	1	36.7882	-2.5815	Bajos Roquetas	Almería	Consejería de Medio Ambiente
16	4	2010	2	36.7015	-2.6432	Cerrillos	Almería	Consejería de Medio Ambiente
16	4	2010	1	36.7431	-2.1187	Genoveses	Almería	Consejería de Medio Ambiente
16	4	2010	3	36.7470	-3.0529	El Lance, Adra	Almería	Consejería de Medio Ambiente
16	4	2010	4	37.2148	-1.8011	El playazo	Almería	Consejería de Medio Ambiente
21	4	2010	3	36.6831	-2.7005	Punta entinas-Sabinal	Almeria	http://www.europapress.es/andalucia/almeria-00350/noticia-retiran-40-ejemplares-medusas-carabela-portuguesa-frente-litoral-punta-entinas-sabinal-20100421173427.html
30	4	2010	1	38.5988	-0.0468	Altea	Alicante	http://www.diarioinformacion.com/alicante/2010/04/30/travesia-peligrosa-carabela/1004284.html
30	4	2010	1	38.5338	-0.1297	Benidorm	Alicante	http://www.diarioinformacion.com/alicante/2010/04/30/travesia-peligrosa-carabela/1004284.html
30	4	2010	1	38.4273	-0.3875	El Campello	Alicante	http://www.diarioinformacion.com/alicante/2010/04/30/travesia-peligrosa-carabela/1004284.html
30	4	2010	1	38.1668	-0.4708	Tabarca	Alicante	http://www.diarioinformacion.com/alicante/2010/04/30/travesia-peligrosa-carabela/1004284.html
30	4	2010	1	37.5583	-1.2692	Mazarrón	Murcia	http://www.laopiniondemurcia.es/municipios/2010/04/30/avistan-decena-carabelas-portuguesas-costa-murciana/244216.html
30	4	2010	1	38.1872	-0.5545	Puerto	Alicante	http://www.diarioinformacion.com/alicante/2010/04/30/travesia-peligrosa-carabela/1004284.html
4	5	2010	1	38.2603	-0.5166	Arenales del Sol	Alicante	http://www.diarioinformacion.com/elche/2010/05/04/temida-carabela-deja-huella-playa-arenales/1005493.html
8	5	2010	1	37.3999	-1.5806	Aguilas	Murcia	http://www.murcia.com/aguilas/noticias/2010/05-08-ava-alerta-sobre-llegada-a.asp
29	5	2010	4	37.0600	-8.1000	Albufeira	Portugal	http://jellywatch.org/sightings_list
12	6	2010	3	38.3410	-0.4611	Coast	Alicante	http://www.gentevalencia.com/noticias/medusas-peligrosas-en-las-costas-valencianas/870/
21	6	2010	3	36.0101	-5.3381	Strait of Gibraltar	Ceuta	http://www.efeverde.com/esl/contenidos/noticias/21-junio-2010-13-12-00-medusa-peligrosa-en-las-aguas-del-estrecho-aunque-lejos-de-la-costa
9	7	2010	Nan	38.2603	-0.5166	Arenales del Sol	Alicante	MMA
11	7	2010	2	43.3199	-1.9869	La Concha	Guipuzcoa	MMA
11	7	2010	3	43.2878	-2.1707	Zarautz	Guipuzcoa	MMA
12	7	2010	1	43.3199	-1.9869	La Concha	Guipuzcoa	http://www.diariovasco.com/v/20100717/al-dia-local/detectan-concha-hondarribia-zarautz-20100717.html
12	7	2010	2	43.3199	-1.9869	La Concha	Guipuzcoa	MMA
13	7	2010	1	43.3805	-1.7937	Hondarribia	Guipuzcoa	http://www.diariovasco.com/v/20100717/al-dia-local/detectan-concha-hondarribia-zarautz-20100717.html
13	7	2010	2	43.3805	-1.7937	Hondarribia	Guipuzcoa	MMA
13	7	2010	1	43.2878	-2.1707	Zarautz	Guipuzcoa	http://www.diariovasco.com/v/20100717/al-dia-local/detectan-concha-hondarribia-zarautz-20100717.html
14	7	2010	1	43.3010	-2.2616	Zumaia	Guipuzcoa	MMA
15	7	2010	3	43.3805	-1.7937	Hondarribia	Guipuzcoa	http://www.diariovasco.com/v/20100717/al-dia-local/detectan-concha-hondarribia-zarautz-20100717.html
15	7	2010	3	43.3805	-1.7937	Hondarribia	Guipuzcoa	MMA
15	7	2010	3	43.3015	-2.2018	Malkorbe, Getaria	Guipuzcoa	MMA
17	7	2010	2	43.3010	-2.2616	Zumaia	Guipuzcoa	MMA
18	7	2010	1	43.4223	-2.7129	Bermeo	Vizcaya	MMA
20	7	2010	Nan	43.3001	-2.3522	Deba, Deba	Guipuzcoa	MMA
20	7	2010	Nan	43.3010	-2.2616	Zumaia	Guipuzcoa	MMA
20	7	2010	Nan	43.2902	-2.1289	La Antilla, Orio	Guipuzcoa	MMA
20	7	2010	4	43.3199	-1.9869	La Concha	Guipuzcoa	MMA
20	7	2010	Nan	43.3205	-2.0003	San Sebastián	Guipuzcoa	MMA
20	7	2010	Nan	43.3015	-2.2018	Malkorbe Getaria	Guipuzcoa	MMA
20	7	2010	1	43.3113	-2.3799	Mutriku	Guipuzcoa	MMA
20	7	2010	Nan	43.3191	-2.0020	Ondarreta	Guipuzcoa	MMA
20	7	2010	Nan	43.2878	-2.1707	Zarautz	Guipuzcoa	MMA
21	7	2010	1	38.5338	-0.1297	Benidorm	Alicante	MMA
21	7	2010	1	43.4651	-3.7264	Loredo	Cantabria	MMA
23	7	2010	4	43.3001	-2.3522	Deba, Deba	Guipuzcoa	MMA
23	7	2010	1	43.2902	-2.1289	La Antilla, Orio	Guipuzcoa	MMA
23	7	2010	1	43.5074	-3.5786	Arnuero	Guipuzcoa	MMA
23	7	2010	2	43.4782	-3.6893	Langre 1	Cantabria	MMA
23	7	2010	1	43.4651	-3.7264	Loredo	Cantabria	MMA
23	7	2010	5	43.3092	-2.3801	Port of Mutriku	Guipuzcoa	MMA
24	7	2010	5	43.3010	-2.2616	Zumaia	Guipuzcoa	MMA
24	7	2010	1	38.7096	0.1681	Benitachell	Alicante	MMA
24	7	2010	2	43.4943	-3.5290	Ris, Noja	Cantabria	MMA
25	7	2010	1	43.3812	-3.2138	Castro Urdiales	Cantabria	MMA
25	7	2010	1	43.4336	-2.8050	Bakio	Vizcaya	MMA
26	7	2010	3	43.5010	-3.6130	Bareyo	Cantabria	MMA
26	7	2010	3	43.3566	-3.0219	Arrigunaga	Vizcaya	MMA
26	7	2010	3	43.3902	-2.9977	Atxabiribil	Vizcaya	MMA
26	7	2010	Nan	43.3817	-3.0200	Azkorri, Getxo	Vizcaya	MMA
26	7	2010	Nan	43.4336	-2.8050	Bakio, Bakio	Vizcaya	MMA
26	7	2010	2	43.3914	-4.2862	Comillas, Comillas	Cantabria	MMA
26	7	2010	3	43.4703	-3.7742	El Camello, Santander	Cantabria	MMA
26	7	2010	3	43.3795	-3.2006	El Pocillo, Castro Urdiales	Cantabria	MMA
26	7	2010	Nan	43.4157	-2.9481	Gorliz, Gorliz	Vizcaya	MMA
26	7	2010	Nan	43.3636	-2.4999	Isuntza, Lekeitio	Vizcaya	MMA
26	7	2010	Nan	43.3010	-2.2616	Zumaia	Guipuzcoa	MMA
26	7	2010	Nan	43.3643	-2.4930	Karraspio, Mendexa	Vizcaya	MMA
26	7	2010	Nan	43.3515	-3.1181	La Arena, Muskiz	Vizcaya	MMA
26	7	2010	3	43.3199	-1.9869	La Concha, San Sebastian	Guipuzcoa	MMA
26	7	2010	1	43.4372	-4.0410	La Concha, Suances	Cantabria	MMA
26	7	2010	Nan	43.3291	-1.9770	La Zurriola, Donostia	Guipuzcoa	MMA
26	7	2010	Nan	43.4110	-2.6584	Laga, Ibarrangelu	Vizcaya	MMA
26	7	2010	Nan	43.4089	-2.6844	Laida, Ibarrangelu	Vizcaya	MMA
26	7	2010	1	43.4782	-3.6893	Ribamontan al Mar	Cantabria	MMA
26	7	2010	3	43.3965	-4.2206	Alfoz de Lloredo	Cantabria	MMA
26	7	2010	Nan	43.3766	-2.5433	Ogeia, Ispaster	Vizcaya	MMA
26	7	2010	Nan	43.3191	-2.0020	Ondarreta, Donostia	Guipuzcoa	MMA
26	7	2010	Nan	43.4134	-2.9485	Plentzia, Plentzia	Vizcaya	MMA
26	7	2010	3	43.4376	-3.4514	San Martín, Santoña	Cantabria	MMA
26	7	2010	3	43.4744	-3.7794	Sardinero 1 Santander	Cantabria	MMA
26	7	2010	1	43.4605	-3.7341	Somo, Ribamontan al Mar	Cantabria	MMA
26	7	2010	3	43.4406	-4.0007	Usgo, Miengo	Cantabria	MMA
26	7	2010	3	43.4777	-3.8753	Virgen del Mar, Santander	Cantabria	MMA
26	7	2010	5	43.2878	-2.1707	Zarautz	Guipuzcoa	MMA
27	7	2010	2	43.4234	-3.4355	La Salve, Laredo	Cantabria	MMA
27	7	2010	1	43.4864	-3.7870	Mataleñas, Santander	Cantabria	MMA
27	7	2010	Nan	43.4943	-3.5290	Ris, Noja	Cantabria	MMA
27	7	2010	2	43.4792	-3.7826	Castañeda, Santander	Cantabria	MMA
28	7	2010	2	43.3066	-2.2062	Getaria	Guipuzcoa	MMA
28	7	2010	Nan	43.4792	-3.7826	Castañeda, Santander	Cantabria	MMA
29	7	2010	3	43.5010	-3.6130	Ajo / Cuberris, Bareyo	Cantabria	MMA
29	7	2010	1	43.3752	-3.2107	Brazomar, Castro Urdiales	Cantabria	MMA
29	7	2010	1	43.4960	-3.5372	Joyel, Noja	Cantabria	MMA
29	7	2010	1	43.4813	-5.2237	La Isla, Colunga	Asturias	MMA
29	7	2010	1	43.5096	-5.2684	Lastres, Colunga	Asturias	MMA
29	7	2010	2	43.3873	-3.2240	Ostende / Urdiales	Cantabria	MMA
29	7	2010	1	43.5074	-3.5786	Ría de Isla, Arnuero	Cantabria	MMA
29	7	2010	1	43.4943	-3.5290	Ris, Noja	Cantabria	MMA
29	7	2010	2	43.5378	-5.3802	Rodiles, Villaviciosa	Asturias	MMA
29	7	2010	Nan	43.4169	-3.3317	Sonabia, Castro Urdiales	Cantabria	MMA
29	7	2010	1	43.4178	-4.7409	Toró, Llanes	Asturias	MMA
30	7	2010	2	43.4378	-4.8248	Barro, Llanes	Asturias	MMA
30	7	2010	2	43.4368	-4.8099	Borizú, Llanes	Asturias	MMA
30	7	2010	Nan	43.4740	-3.9104	Covachos, Bezana	Cantabria	MMA
30	7	2010	Nan	43.4703	-3.7742	El Camello, Santander	Cantabria	MMA
30	7	2010	Nan	43.3795	-3.2006	El Pocillo, Castro Urdiales	Cantabria	MMA
30	7	2010	Nan	43.3831	-3.2114	El Rompeolas	Cantabria	MMA
30	7	2010	Nan	43.5074	-3.5786	Isla, Arnuero	Cantabria	MMA
30	7	2010	Nan	43.4726	-3.7787	La Concha, Santander	Cantabria	MMA
30	7	2010	1	43.3959	-4.5732	La Franca, Ribadeva	Asturias	MMA
30	7	2010	2	43.4234	-3.4355	La Salvé, Laredo	Cantabria	MMA

30	7	2010	Nan	43.4782	-3.6893	Ribamontan al Mar	Cantabria	MMA
30	7	2010	Nan	43.4651	-3.7264	Loredo, Ribamontan al Mar	Cantabria	MMA
30	7	2010	Nan	43.4667	-3.7690	Los Bikinis, Santander	Cantabria	MMA
30	7	2010	Nan	43.4641	-3.7781	Los Peligros, Santander	Cantabria	MMA
30	7	2010	Nan	43.4667	-3.7740	Magdalena, Santander	Cantabria	MMA
30	7	2010	Nan	43.4864	-3.7870	Mataleñas, Santander	Cantabria	MMA
30	7	2010	Nan	43.3873	-3.2240	Ostende / Urdiales	Cantabria	MMA
30	7	2010	Nan	43.3974	-4.3282	Oyambre, Valdaliga	Cantabria	MMA
30	7	2010	Nan	43.4749	-3.7824	Piquio, Santander	Cantabria	MMA
30	7	2010	Nan	43.4943	-3.5290	Ris, Noja	Cantabria	MMA
30	7	2010	Nan	43.4745	-3.8910	San Juan de la Canal	Cantabria	MMA
30	7	2010	2	43.4338	-4.7889	San Martín, Llanes	Asturias	MMA
30	7	2010	Nan	43.4744	-3.7794	Sardinero 1, Santander	Cantabria	MMA
30	7	2010	Nan	43.4605	-3.7341	Somo, Ribamontan al Mar	Cantabria	MMA
30	7	2010	2	43.4423	-4.8372	Toranda, Llanes	Asturias	MMA
30	7	2010	Nan	43.4768	-3.4768	Tregandin, Noja	Cantabria	MMA
31	7	2010	Nan	43.4573	-3.9623	Canallave, Pielagos	Cantabria	MMA
31	7	2010	1	40.4056	-3.2659	Cerdigo, Castro Urdiales	Cantabria	MMA
31	7	2010	Nan	43.4703	-3.7742	El Camello, Santander	Cantabria	MMA
31	7	2010	Nan	43.4601	-3.7591	El Puntal, Santander	Cantabria	MMA
31	7	2010	Nan	43.4989	-3.5401	El Sable, Arnuero	Cantabria	MMA
31	7	2010	2	43.4981	-3.5401	Estaño, Gijón	Asturias	MMA
31	7	2010	5	43.3805	-1.7937	Hondarribia	Guipuzcoa	MMA
31	7	2010	Nan	43.4726	-3.7787	La Concha, Santander	Cantabria	MMA
31	7	2010	1	43.4785	-5.2122	La Espasa, Caravia	Asturias	MMA
31	7	2010	1	43.4813	-5.2237	La Isla, Colunga	Asturias	MMA
31	7	2010	Nan	43.4234	-3.4355	La Salve, Laredo	Cantabria	MMA
31	7	2010	1	43.4136	-3.3697	Liendo, Liendo	Cantabria	MMA
31	7	2010	Nan	43.4667	-3.7690	Los Bikinis, Santander	Cantabria	MMA
31	7	2010	2	43.4769	-5.1775	Morris, Caravia	Asturias	MMA
31	7	2010	1	43.5495	-5.5980	Ñora, Villaviciosa	Asturias	MMA
31	7	2010	Nan	43.4075	-3.3194	Oriñón, Castro Urdiales	Cantabria	MMA
31	7	2010	Nan	43.3974	-4.3282	Oyambre, Valdaliga	Cantabria	MMA
31	7	2010	1	43.5378	-5.3802	Rodiles, Villaviciosa	Asturias	MMA
31	7	2010	Nan	43.4376	-3.4514	San Martín, Santoña	Cantabria	MMA
31	7	2010	Nan	43.4744	-3.7794	Sardinero 1, Santander	Cantabria	MMA
31	7	2010	Nan	43.4605	-3.7341	Somo, Ribamontan al Mar	Cantabria	MMA
31	7	2010	Nan	43.4777	-3.8753	Virgen del Mar, Santander	Cantabria	MMA
1	8	2010	Nan	43.4740	-3.9104	Covachos, Bezana	Cantabria	MMA
1	8	2010	Nan	43.4703	-3.7742	El Camello, Santander	Cantabria	MMA
1	8	2010	Nan	43.4601	-3.7591	El Puntal	Cantabria	MMA
1	8	2010	Nan	43.3864	-4.3889	San Vicente de la Barquera	Cantabria	MMA
1	8	2010	1	43.4731	-3.7558	Isla Mouro / Cabo de Lata	Cantabria	MMA
1	8	2010	Nan	43.5074	-3.5786	La Arena, Arnuero	Cantabria	MMA
1	8	2010	Nan	43.4726	-3.7787	La Concha, Santander	Cantabria	MMA
1	8	2010	Nan	43.4234	-3.4355	La Salve, Laredo	Cantabria	MMA
1	8	2010	Nan	43.4782	-3.6893	Ribamontan al Mar	Cantabria	MMA
1	8	2010	Nan	43.4651	-3.7264	Loredo, Ribamontan al Mar	Cantabria	MMA
1	8	2010	Nan	43.4667	-3.7740	Magdalena, Santander	Cantabria	MMA
1	8	2010	Nan	43.4075	-3.3194	Oriñón, Castro Urdiales	Cantabria	MMA
1	8	2010	Nan	43.3873	-3.2240	Ostende / Urdiales	Cantabria	MMA
1	8	2010	Nan	43.3974	-4.3282	Oyambre, Valdaliga	Cantabria	MMA
1	8	2010	Nan	43.4744	-3.7794	Sardinero 1, Santander	Cantabria	MMA
1	8	2010	Nan	43.4605	-3.7341	Somo, Ribamontan al Mar	Cantabria	MMA
1	8	2010	Nan	43.4280	-4.0950	Tagle / El Sable, Suances	Cantabria	MMA
2	8	2010	2	43.3812	-3.2138	Bahía, Castro Urdiales	Cantabria	MMA
2	8	2010	1	43.5532	-6.3696	Cadavedo, Valdés	Asturias	MMA
2	8	2010	2	43.4573	-3.9623	Canallave, Pielagos	Cantabria	MMA
2	8	2010	1	43.4703	-3.7742	El Camello, Santander	Cantabria	MMA
2	8	2010	1	43.4834	-3.8348	La Maruca, Santander	Cantabria	MMA
2	8	2010	Nan	43.4234	-3.4355	La Salve, Laredo	Cantabria	MMA
2	8	2010	1	43.4667	-3.7690	Los Bikinis, Santander	Cantabria	MMA
2	8	2010	1	43.4391	-4.0484	Los Locos, Suances	Cantabria	MMA
2	8	2010	1	43.4641	-3.7781	Los Peligros, Santander	Cantabria	MMA
2	8	2010	1	43.4667	-3.7740	Magdalena, Santander	Cantabria	MMA
2	8	2010	2	43.3873	-3.2240	Ostende / Urdiales	Cantabria	MMA
2	8	2010	1	43.3854	-3.2142	Peñón de Santa Ana	Cantabria	MMA
2	8	2010	2	43.4943	-3.5290	Ris, Noja	Cantabria	MMA
3	8	2010	3	43.5010	-3.6130	Ajo / Cuberris, Bareyo	Cantabria	MMA
3	8	2010	3	43.3812	-3.2138	Bahía, Castro Urdiales	Cantabria	MMA
3	8	2010	4	43.4336	-2.8050	Bakio	Vizcaya	MMA
3	8	2010	Nan	43.4713	-3.4631	Berria, Santoña	Cantabria	MMA
3	8	2010	2	43.3914	-4.2862	Comillas, Comillas	Cantabria	MMA
3	8	2010	2	43.4703	-3.7742	El Camello, Santander	Cantabria	MMA
3	8	2010	1	43.4601	-3.7591	Ribamontan al Mar	Cantabria	MMA
3	8	2010	2	43.4744	-3.7794	El Sardinero 1, Santander	Cantabria	MMA
3	8	2010	Nan	43.4234	-3.4355	La Salve, Laredo	Cantabria	MMA
3	8	2010	1	43.4860	-5.1451	La Vega, Ribadesella	Asturias	MMA
3	8	2010	4	43.4782	-3.6893	Langre, Ribamontan al Mar	Cantabria	MMA
3	8	2010	4	43.4651	-3.7264	Loredo, Ribamontan al Mar	Cantabria	MMA
3	8	2010	2	43.4391	-4.0484	Los Locos, Suances	Cantabria	MMA
3	8	2010	4	43.3965	-4.2206	Luaña / Cobreces	Cantabria	MMA
3	8	2010	2	43.4864	-3.7870	Mataleñas, Santander	Cantabria	MMA
3	8	2010	1	43.3941	-4.3803	San Vicente de la Barquera	Cantabria	MMA
3	8	2010	3	43.5453	-5.4940	Merón, Villaviciosa	Asturias	MMA
3	8	2010	4	43.3873	-3.2240	Ostende / Urdiales	Cantabria	MMA
3	8	2010	2	43.4282	-4.0963	Santillana del Mar	Cantabria	MMA
3	8	2010	4	43.4605	-3.7341	Somo, Ribamontan al Mar	Cantabria	MMA
3	8	2010	4	43.4280	-4.0950	Tagle / El Sable, Suances	Cantabria	MMA
3	8	2010	2	43.4571	-3.9730	Valdearenas, Piélagos	Cantabria	MMA
3	8	2010	4	43.3994	-4.3668	San Vicente de la Barquera	Cantabria	MMA
3	8	2010	2	43.4777	-3.8753	Virgen del Mar, Santander	Cantabria	MMA
3	8	2010	1	43.3048	-2.2516	Zumaia	Guipuzcoa	MMA
4	8	2010	2	43.4132	-4.7067	Andrín, Llanes	Asturias	MMA
4	8	2010	2	43.4573	-3.9623	Canallave, Pielagos	Cantabria	MMA
4	8	2010	Nan	43.4740	-3.9104	Covachos, Bezana	Cantabria	MMA
4	8	2010	1	43.4601	-3.7591	Ribamontan al Mar	Cantabria	MMA
4	8	2010	Nan	43.3948	-4.4693	El Sable, Arnuero	Cantabria	MMA
4	8	2010	2	43.4821	-3.6782	Galizano	Cantabria	MMA
4	8	2010	1	43.3452	-3.0175	Getxo	Vizcaya	MMA
4	8	2010	Nan	43.4726	-3.7787	La Concha, Santander	Cantabria	MMA
4	8	2010	4	43.4782	-3.6893	Langre, Ribamontan al Mar	Cantabria	MMA
4	8	2010	4	43.4651	-3.7264	Loredo, Ribamontan al Mar	Cantabria	MMA
4	8	2010	4	43.4391	-4.0484	Los Locos, Suances	Cantabria	MMA
4	8	2010	Nan	43.3965	-4.2206	Luaña / Cobreces	Cantabria	MMA
4	8	2010	3	43.5453	-5.4940	Merón, Villaviciosa	Asturias	MMA
4	8	2010	3	43.3873	-3.2240	Ostende / Urdiales	Cantabria	MMA
4	8	2010	1	43.4223	-4.7495	Sablón, Llanes	Asturias	MMA
4	8	2010	2	43.4605	-3.7341	Somo, Ribamontan al Mar	Cantabria	MMA
4	8	2010	4	43.4280	-4.0950	Tagle / El Sable, Suances	Cantabria	MMA
4	8	2010	1	43.4455	-4.8522	Torimbia, Llanes	Asturias	MMA
4	8	2010	2	43.4571	-3.9730	Valdearenas, Piélagos	Cantabria	MMA
4	8	2010	3	43.4035	-4.6545	Vidiago, Llanes	Asturias	MMA
4	8	2010	2	43.4777	-3.8753	Virgen del Mar, Santander	Cantabria	MMA
5	8	2010	1	43.5010	-3.6130	Ajo / Cuberris, Bareyo	Cantabria	MMA
5	8	2010	2	43.6081	-7.3017	Arealonga, Foz	Lugo	MMA
5	8	2010	1	43.3812	-3.2138	Bahía, Castro Urdiales	Cantabria	MMA
5	8	2010	1	43.4378	-4.8248	Barro, Llanes	Asturias	MMA

5	8	2010	1	43.3752	-3.2107	Brazomar, Castro Urdiales	Cantabria	MMA
5	8	2010	2	43.4573	-3.9623	Canallave, Pielagos	Cantabria	MMA
5	8	2010	2	43.4703	-3.7742	El Camello, Santander	Cantabria	MMA
5	8	2010	4	43.4601	-3.7591	Ribamontan al Mar	Cantabria	MMA
5	8	2010	Nan	43.4989	-3.5401	El Sable, Arnuero	Cantabria	MMA
5	8	2010	2	43.5477	-5.5294	España, Villaviciosa	Asturias	MMA
5	8	2010	3	43.4821	-3.6782	Galizano	Cantabria	MMA
5	8	2010	1	43.5074	-3.5786	La Arena, Arnuero	Cantabria	MMA
5	8	2010	1	43.4726	-3.7787	La Concha, Santander	Cantabria	MMA
5	8	2010	1	43.4372	-4.0410	La Concha, Suances	Cantabria	MMA
5	8	2010	4	43.4834	-3.8348	La Maruca, Santander	Cantabria	MMA
5	8	2010	2	43.4234	-3.4355	La Salve, Laredo	Cantabria	MMA
5	8	2010	2	43.4860	-5.1451	La Vega, Ribadesella	Asturias	MMA
5	8	2010	2	43.4782	-3.6893	Langre, Ribamontan al Mar	Cantabria	MMA
5	8	2010	1	43.4651	-3.7264	Loredo, Ribamontan al Mar	Cantabria	MMA
5	8	2010	1	43.6185	-5.7854	Luanco, Gozón	Asturias	MMA
5	8	2010	2	43.3965	-4.2206	Luaña / Cobreces	Cantabria	MMA
5	8	2010	Nan	43.3941	-4.3803	San Vicente de la Barquera	Cantabria	MMA
5	8	2010	2	43.3974	-4.3282	Oyambre, Valdaliga	Cantabria	MMA
5	8	2010	1	43.6626	-7.3518	Portocelo/Urbana, Burela	Lugo	MMA
5	8	2010	2	43.5735	-7.2455	Rapadoira, Foz	Lugo	MMA
5	8	2010	2	43.4338	-4.7889	San Martín, Llanes	Asturias	MMA
5	8	2010	1	43.4731	-3.7297	Ribamontan al Mar	Cantabria	MMA
5	8	2010	2	43.4605	-3.7341	Somo, Ribamontan al Mar	Cantabria	MMA
5	8	2010	2	43.4280	-4.0950	Tagle / El Sable, Suances	Cantabria	MMA
5	8	2010	2	43.4423	-4.8372	Toranda, Llanes	Asturias	MMA
5	8	2010	5	43.3947	-4.4872	Val de San Vicente, Pechón	Cantabria	MMA
5	8	2010	4	43.4571	-3.9730	Valdearenas, Piélagos	Cantabria	MMA
5	8	2010	4	43.4777	-3.8753	Virgen del Mar, Santander	Cantabria	MMA
6	8	2010	Nan	43.4748	-3.9140	Arnia, Piélagos	Cantabria	MMA
6	8	2010	1	43.6324	-5.8090	Bañugues, Gozón	Asturias	MMA
6	8	2010	Nan	43.4703	-3.7742	El Camello, Santander	Cantabria	MMA
6	8	2010	Nan	43.4601	-3.7591	Ribamontan al Mar	Cantabria	MMA
6	8	2010	Nan	43.3864	-4.3889	San Vicente de la Barquera	Cantabria	MMA
6	8	2010	3	43.5049	-3.6450	Santoña	Cantabria	MMA
6	8	2010	3	43.4731	-3.7558	Isla Mouro / Cabo de Lata	Cantabria	MMA
6	8	2010	1	43.4649	-5.0543	La Atalaya, Ribadesella	Asturias	MMA
6	8	2010	Nan	43.4726	-3.7787	La Concha, Santander	Cantabria	MMA
6	8	2010	Nan	43.4320	-4.0341	La Ribera, Suances	Cantabria	MMA
6	8	2010	Nan	43.4234	-3.4355	La Salve, Laredo	Cantabria	MMA
6	8	2010	Nan	43.4782	-3.6893	Ribamontan al Mar	Cantabria	MMA
6	8	2010	Nan	43.4667	-3.7690	Los Bikinis, Santander	Cantabria	MMA
6	8	2010	Nan	43.3965	-4.2206	Luaña / Cobreces	Cantabria	MMA
6	8	2010	Nan	43.4667	-3.7740	Magdalena, Santander	Cantabria	MMA
6	8	2010	Nan	43.4864	-3.7870	Mataleñas, Santander	Cantabria	MMA
6	8	2010	Nan	43.4075	-3.3194	Oriñón, Castro Urdiales	Cantabria	MMA
6	8	2010	Nan	43.3974	-4.3282	Oyambre, Valdaliga	Cantabria	MMA
6	8	2010	Nan	43.4749	-3.7824	Piquio, Santander	Cantabria	MMA
6	8	2010	Nan	43.4744	-3.7794	Sardinero 1, Santander	Cantabria	MMA
6	8	2010	Nan	43.4792	-3.7826	Sardinero 2 / Castañeda	Cantabria	MMA
7	8	2010	1	43.4748	-3.9140	Arnia, Piélagos	Cantabria	MMA
7	8	2010	Nan	43.3914	-4.2862	Comillas, Comillas	Cantabria	MMA
7	8	2010	3	43.3001	-2.3522	Deba	Guipuzcoa	MMA
7	8	2010	2	43.4703	-3.7742	El Camello, Santander	Cantabria	MMA
7	8	2010	3	43.3364	-2.0850	Guipuzcoa	Guipuzcoa	MMA
7	8	2010	2	43.4731	-3.7558	Isla Mouro / Cabo de Lata	Cantabria	MMA
7	8	2010	2	43.4234	-3.4355	La Salve, Laredo	Cantabria	MMA
7	8	2010	1	43.4782	-3.6893	Langre, Ribamontan al Mar	Cantabria	MMA
7	8	2010	1	43.4651	-3.7264	Loredo, Ribamontan al Mar	Cantabria	MMA
7	8	2010	Nan	43.3965	-4.2206	Luaña / Cobreces	Cantabria	MMA
7	8	2010	2	43.4667	-3.7740	Magdalena, Santander	Cantabria	MMA
7	8	2010	3	43.3015	-2.2018	Malkorbe, Getaria	Guipuzcoa	MMA
7	8	2010	1	43.3654	-3.1925	Castro Urdiales	Cantabria	MMA
7	8	2010	1	43.4075	-3.3194	Oriñón, Castro Urdiales	Cantabria	MMA
7	8	2010	1	43.3974	-4.3282	Oyambre, Valdaliga	Cantabria	MMA
7	8	2010	1	43.4749	-3.7824	Piquio, Santander	Cantabria	MMA
7	8	2010	1	43.4745	-3.8910	San Juan de la Canal	Cantabria	MMA
7	8	2010	Nan	43.4792	-3.7826	Sardinero 2	Cantabria	MMA
7	8	2010	1	43.4605	-3.7341	Somo, Ribamontan al Mar	Cantabria	MMA
7	8	2010	1	43.4169	-3.3317	Sonabia, Castro Urdiales	Cantabria	MMA
8	8	2010	1	43.4713	-3.4631	Berria, Santoña	Cantabria	MMA
8	8	2010	1	43.3001	-2.3522	Deba	Guipuzcoa	MMA
8	8	2010	2	43.5049	-3.5449	El Ardanal, Arnuero	Cantabria	MMA
8	8	2010	1	43.4703	-3.7742	El Camello, Santander	Cantabria	MMA
8	8	2010	2	43.4989	-3.5401	El Sable, Arnuero	Cantabria	MMA
8	8	2010	1	43.3066	-2.2062	Getaria	Guipuzcoa	MMA
8	8	2010	1	43.5074	-3.5786	La Arena, Arnuero	Cantabria	MMA
8	8	2010	1	43.4234	-3.4355	La Salve, Laredo	Cantabria	MMA
8	8	2010	1	43.4667	-3.7690	Los Bikinis, Santander	Cantabria	MMA
8	8	2010	1	43.4641	-3.7781	Los Peligros, Santander	Cantabria	MMA
8	8	2010	1	43.4667	-3.7740	Magdalena, Santander	Cantabria	MMA
8	8	2010	1	43.4444	-3.9941	Miengo	Cantabria	MMA
8	8	2010	1	43.3873	-3.2240	Ostende / Urdiales	Cantabria	MMA
8	8	2010	1	43.3974	-4.3282	Oyambre, Valdaliga	Cantabria	MMA
8	8	2010	2	43.4749	-3.7824	Piquio, Santander	Cantabria	MMA
8	8	2010	2	43.4792	-3.7826	Sardinero 2	Cantabria	MMA
9	8	2010	4	43.4039	-3.3133	Arenillas, Castro Urdiales	Cantabria	MMA
9	8	2010	1	43.4378	-4.8248	Barro, Llanes	Asturias	MMA
9	8	2010	2	43.3752	-3.2107	Brazomar, Castro Urdiales	Cantabria	MMA
9	8	2010	5	43.3293	-1.9943	Donostia - San Sebastian	Guipuzcoa	MMA
9	8	2010	4	43.4703	-3.7742	El Camello, Santander	Cantabria	MMA
9	8	2010	1	43.4223	-4.7495	El Sablón, Llanes	Asturias	MMA
9	8	2010	2	43.4731	-3.7558	Isla Mouro / Cabo de Lata	Cantabria	MMA
9	8	2010	5	43.3809	-2.5250	Ispaster - Lekeitio	Vizcaya	MMA
9	8	2010	2	43.4726	-3.7787	La Concha, Santander	Cantabria	MMA
9	8	2010	1	43.5084	-5.2595	La Griega, Colunga	Asturias	MMA
9	8	2010	3	43.4864	-3.7870	Mataleñas, Santander	Cantabria	MMA
9	8	2010	Nan	43.3941	-4.3803	Merón	Cantabria	MMA
9	8	2010	2	43.3654	-3.1925	Mioño / Dicido	Cantabria	MMA
9	8	2010	2	43.3873	-3.2240	Ostende / Urdiales	Cantabria	MMA
9	8	2010	1	43.4749	-3.7824	Piquio, Santander	Cantabria	MMA
9	8	2010	1	43.4745	-3.8910	San Juan de la Canal	Cantabria	MMA
9	8	2010	4	43.4744	-3.7794	Sardinero 1, Santander	Cantabria	MMA
9	8	2010	1	43.3994	-4.3668	Vederna	Cantabria	MMA
10	8	2010	2	43.5010	-3.6130	Ajo / Cuberris, Bareyo	Cantabria	MMA
10	8	2010	4	43.4039	-3.3133	Arenillas, Castro Urdiales	Cantabria	MMA
10	8	2010	1	43.4137	-4.7114	Ballota, Llanes	Asturias	MMA
10	8	2010	1	43.4378	-4.8248	Barro, Llanes	Asturias	MMA
10	8	2010	4	43.4713	-3.4631	Berria, Santoña	Cantabria	MMA
10	8	2010	4	43.4368	-4.8099	Borizú, Llanes	Asturias	MMA
10	8	2010	3	43.3752	-3.2107	Brazomar, Castro Urdiales	Cantabria	MMA
10	8	2010	5	43.3293	-1.9943	Donostia - San Sebastian	Guipuzcoa	MMA
10	8	2010	2	43.4015	-4.3573	El Cabo / Gerra	Cantabria	MMA
10	8	2010	2	43.4703	-3.7742	El Camello, Santander	Cantabria	MMA
10	8	2010	2	43.3795	-3.2006	El Pocillo, Castro Urdiales	Cantabria	MMA
10	8	2010	3	43.3864	-4.3889	El Rosal	Cantabria	MMA
10	8	2010	1	43.4989	-3.5401	El Sable, Arnuero	Cantabria	MMA
10	8	2010	4	43.4223	-4.7495	El Sablón, Llanes	Asturias	MMA

10	8	2010	Nan	43.4821	-3.6782	Ribamontan al Mar	Cantabria	MMA
10	8	2010	Nan	43.3643	-2.4930	Karraspio, Mendexa	Cantabria	MMA
10	8	2010	1	43.5074	-3.5786	La Arena, Arnuero	Cantabria	MMA
10	8	2010	1	43.4834	-3.8348	La Maruca, Santander	Cantabria	MMA
10	8	2010	Nan	43.4234	-3.4355	La Salve, Laredo	Cantabria	MMA
10	8	2010	3	43.4667	-3.7690	Los Bikinis, Santander	Cantabria	MMA
10	8	2010	2	43.4641	-3.7781	Los Peligros, Santander	Cantabria	MMA
10	8	2010	4	43.4864	-3.7870	Mataleñas, Santander	Cantabria	MMA
10	8	2010	Nan	43.3517	-2.4746	Mendexa, Lekeitio	Vizcaya	MMA
10	8	2010	Nan	43.3941	-4.3803	Merón	Cantabria	MMA
10	8	2010	3	43.3654	-3.1925	Mioño / Dicido	Cantabria	MMA
10	8	2010	2	43.4835	-3.7843	Molinucos, Santander	Cantabria	MMA
10	8	2010	4	43.4075	-3.3194	Oriñón, Castro Urdiales	Cantabria	MMA
10	8	2010	2	43.3873	-3.2240	Ostende / Urdiales	Cantabria	MMA
10	8	2010	3	43.3974	-4.3282	Oyambre, Valdaliga	Cantabria	MMA
10	8	2010	2	43.4332	-4.7854	Póo, Llanes	Asturias	MMA
10	8	2010	2	43.5604	-5.4031	Punta del Olivo / Tazones	Asturias	MMA
10	8	2010	2	43.4943	-3.5290	Ris, Noja	Cantabria	MMA
10	8	2010	4	43.3831	-3.2114	Rompeolas, Castro Urdiales	Cantabria	MMA
10	8	2010	Nan	43.4376	-3.4514	San Martín, Santoña	Cantabria	MMA
10	8	2010	Nan	43.4768	-3.4768	Tregandin, Noja	Cantabria	MMA
10	8	2010	3	43.4406	-4.0007	Usgo, Miengo	Cantabria	MMA
10	8	2010	Nan	43.4571	-3.9730	Valdearenas, Piélagos	Cantabria	MMA
11	8	2010	2	43.4132	-4.7067	Andrín, Llanes	Asturias	MMA
11	8	2010	1	43.4617	-4.9355	Cuevas del Mar, Llanes	Asturias	MMA
11	8	2010	5	43.3293	-1.9943	Donostia - San Sebastian	Guipuzcoa	MMA
11	8	2010	2	43.4223	-4.7495	El Sablón, Llanes	Asturias	MMA
11	8	2010	2	43.4792	-3.7826	El Sardinero 2	Cantabria	MMA
11	8	2010	2	43.4197	-4.7441	Puerto Chicu y Toró	Asturias	MMA
11	8	2010	4	43.3636	-2.4999	Isuntza, Lekeitio	Vizcaya	MMA
11	8	2010	1	43.4860	-5.1451	La Vega, Ribadesella	Asturias	MMA
11	8	2010	2	43.4651	-3.7264	Loredo, Ribamontan al Mar	Cantabria	MMA
11	8	2010	2	43.4391	-4.0484	Los Locos, Suances	Cantabria	MMA
11	8	2010	2	43.4667	-3.7740	Magdalena, Santander	Cantabria	MMA
11	8	2010	1	43.3873	-3.2240	Ostende / Urdiales	Cantabria	MMA
11	8	2010	2	43.3974	-4.3282	Oyambre, Valdaliga	Cantabria	MMA
11	8	2010	2	43.4332	-4.7854	Póo, Llanes	Asturias	MMA
11	8	2010	1	43.4035	-4.6545	Vidiago, Llanes	Asturias	MMA
12	8	2010	Nan	27.7895	-15.7256	Amadores, Mogán	Gran Canaria	MMA
12	8	2010	Nan	43.3947	-4.4872	Amio, Val de San Vicente	Cantabria	MMA
12	8	2010	Nan	43.3817	-3.0200	Azcorri, Getxo	Vizcaya	MMA
12	8	2010	2	43.4573	-3.9623	Canallave, Pielagos	Cantabria	MMA
12	8	2010	2	43.3914	-4.2862	Comillas, Comillas	Cantabria	MMA
12	8	2010	5	43.3293	-1.9943	Donostia - San Sebastian	Guipuzcoa	MMA
12	8	2010	1	43.4015	-4.3573	El Cabo / Gerra	Cantabria	MMA
12	8	2010	1	43.4703	-3.7742	El Camello, Santander	Cantabria	MMA
12	8	2010	3	43.4744	-3.7794	El Sardinero, Santander	Cantabria	MMA
12	8	2010	2	43.4821	-3.6782	Ribamontan al Mar	Cantabria	MMA
12	8	2010	1	43.3941	-4.3806	La Braña	Cantabria	MMA
12	8	2010	2	43.4834	-3.8348	La Maruca, Santander	Cantabria	MMA
12	8	2010	Nan	43.3886	-4.3914	La Maza	Cantabria	MMA
12	8	2010	1	43.4234	-3.4355	La Salve, Laredo	Cantabria	MMA
12	8	2010	2	43.4651	-3.7264	Loredo, Ribamontan al Mar	Cantabria	MMA
12	8	2010	2	43.4391	-4.0484	Los Locos, Suances	Cantabria	MMA
12	8	2010	3	43.4667	-3.7740	Magdalena, Santander	Cantabria	MMA
12	8	2010	3	43.4864	-3.7870	Mataleñas, Santander	Cantabria	MMA
12	8	2010	1	43.3941	-4.3803	Merón	Cantabria	MMA
12	8	2010	3	43.4605	-3.7341	Somo, Ribamontan al Mar	Cantabria	MMA
12	8	2010	3	43.4280	-4.0950	Tagle / El Sable, Suances	Cantabria	MMA
12	8	2010	3	43.4571	-3.9730	Valdearenas, Piélagos	Cantabria	MMA
12	8	2010	3	43.4777	-3.8753	Virgen del Mar, Santander	Cantabria	MMA
13	8	2010	4	43.5010	-3.6130	Ajo / Cuberris, Bareyo	Cantabria	MMA
13	8	2010	4	43.4748	-3.9140	Arnia, Piélagos	Cantabria	MMA
13	8	2010	3	43.4577	-3.8041	Bahía de Santander	Cantabria	MMA
13	8	2010	2	43.4573	-3.9623	Canallave, Pielagos	Cantabria	MMA
13	8	2010	5	43.3293	-1.9943	Donostia - San Sebastian	Cantabria	MMA
13	8	2010	4	43.4015	-4.3573	El Cabo / Gerra	Cantabria	MMA
13	8	2010	1	43.4744	-3.7794	El Sardinero, Santander	Cantabria	MMA
13	8	2010	1	43.4731	-3.7558	Isla Mouro / Cabo de Lata	Cantabria	MMA
13	8	2010	2	43.4834	-3.8348	La Maruca, Santander	Cantabria	MMA
13	8	2010	2	43.4782	-3.6893	Langre, Ribamontan al Mar	Cantabria	MMA
13	8	2010	4	43.4391	-4.0484	Los Locos, Suances	Cantabria	MMA
13	8	2010	2	43.3654	-3.1925	Mioño / Dicido	Cantabria	MMA
13	8	2010	2	43.4698	-3.9326	Portio, Piélagos	Cantabria	MMA
13	8	2010	3	43.4280	-4.0950	Tagle / El Sable, Suances	Cantabria	MMA
13	8	2010	4	43.4571	-3.9730	Valdearenas, Piélagos	Cantabria	MMA
14	8	2010	Nan	43.5010	-3.6130	Ajo / Cuberris, Bareyo	Cantabria	MMA
14	8	2010	1	43.4341	-2.8934	Armintza	Vizcaya	MMA
14	8	2010	5	43.4577	-3.8041	Bahía de Santander	Cantabria	MMA
14	8	2010	Nan	43.4336	-2.8050	Bakio, Bakio	Vizcaya	MMA
14	8	2010	4	43.4740	-3.9104	Covachos, Bezana	Cantabria	MMA
14	8	2010	3	43.4401	-2.9353	Isla Villano, Armintza	Vizcaya	MMA
14	8	2010	1	43.4726	-3.7787	La Concha, Santander	Cantabria	MMA
14	8	2010	3	43.4834	-3.8348	La Maruca, Santander	Cantabria	MMA
14	8	2010	1	43.4136	-3.3697	Liendo, Liendo	Cantabria	MMA
14	8	2010	2	43.4667	-3.7690	Los Bikinis, Santander	Cantabria	MMA
14	8	2010	3	43.4641	-3.7781	Los Peligros, Santander	Cantabria	MMA
14	8	2010	2	43.4667	-3.7740	Magdalena, Santander	Cantabria	MMA
14	8	2010	1	43.3941	-4.3803	Merón	Cantabria	MMA
14	8	2010	2	43.4613	-3.7944	Puerto Chico, Santander	Cantabria	MMA
14	8	2010	1	43.3994	-4.3668	San Vicente de la Barquera	Cantabria	MMA
14	8	2010	4	43.4777	-3.8753	Virgen del Mar, Santander	Cantabria	MMA
15	8	2010	1	43.4378	-4.8248	Barro, Llanes	Asturias	MMA
15	8	2010	1	43.4368	-4.8099	Borizú, Llanes	Asturias	MMA
15	8	2010	1	43.4740	-3.9104	Covachos, Bezana	Cantabria	MMA
15	8	2010	1	43.4839	-3.8329	El Bocal, Santander	Cantabria	MMA
15	8	2010	1	43.4601	-3.7591	Ribamontan al Mar	Cantabria	MMA
15	8	2010	1	43.4785	-5.2122	La Espasa, Caravia	Asturias	MMA
15	8	2010	2	43.5084	-5.2595	La Griega, Colunga	Asturias	MMA
15	8	2010	4	43.4834	-3.8348	La Maruca, Santander	Cantabria	MMA
15	8	2010	3	43.4782	-3.6893	Langre, Ribamontan al Mar	Cantabria	MMA
15	8	2010	2	43.4651	-3.7264	Loredo, Ribamontan al Mar	Cantabria	MMA
15	8	2010	1	43.6185	-5.7854	Luanco, Gozón	Asturias	MMA
15	8	2010	2	43.4769	-5.1775	Morris, Caravia	Asturias	MMA
15	8	2010	4	43.5501	-5.4891	Ñora - Rodiles, Villaviciosa	Asturias	MMA
15	8	2010	2	43.4340	-4.8067	Palombina, Llanes	Asturias	MMA
15	8	2010	1	43.4605	-3.7341	Somo, Ribamontan al Mar	Cantabria	MMA
15	8	2010	4	43.4687	-3.9460	Somocuevas, Piélagos	Cantabria	MMA
15	8	2010	3	43.4280	-4.0950	Tagle / El Sable, Suances	Cantabria	MMA
15	8	2010	1	43.4406	-4.0007	Usgo, Miengo	Cantabria	MMA
16	8	2010	Nan	43.4336	-2.8050	Bakio, Bakio	Vizcaya	MMA
16	8	2010	4	43.3293	-1.9943	Donostia - San Sebastian	Guipuzcoa	MMA
16	8	2010	1	43.4601	-3.7591	Ribamontan al Mar	Cantabria	MMA
16	8	2010	1	43.4989	-3.5401	El Sable, Arnuero	Cantabria	MMA
16	8	2010	4	43.4792	-3.7826	El Sardinero 2	Cantabria	MMA
16	8	2010	Nan	43.4744	-3.7794	El Sardinero, Santander	Cantabria	MMA
16	8	2010	2	43.4821	-3.6782	Ribamontan al Mar	Cantabria	MMA
16	8	2010	2	43.4726	-3.7787	La Concha, Santander	Cantabria	MMA

16	8	2010	3	43.4813	-5.2237	La Isla, Colunga	Asturias	MMA
16	8	2010	Nan	43.4320	-4.0341	La Ribera, Suances	Cantabria	MMA
16	8	2010	3	43.4234	-3.4355	La Salve, Laredo	Cantabria	MMA
16	8	2010	3	43.5096	-5.2684	Lastres, Colunga	Asturias	MMA
16	8	2010	1	43.4641	-3.7781	Los Peligros, Santander	Cantabria	MMA
16	8	2010	1	43.6185	-5.7854	Luanco, Gozón	Asturias	MMA
16	8	2010	Nan	43.4667	-3.7740	Magdalena, Santander	Cantabria	MMA
16	8	2010	1	43.4340	-4.8067	Palombina, Llanes	Asturias	MMA
16	8	2010	1	43.4195	-4.7471	Puertu Chicu, Llanes	Asturias	MMA
16	8	2010	Nan	43.4745	-3.8910	San Juan de la Canal	Cantabria	MMA
16	8	2010	2	43.4338	-4.7889	San Martín, Llanes	Asturias	MMA
16	8	2010	3	43.3315	-3.0270	Santurtzi, Santurtzi	Vizcaya	MMA
16	8	2010	1	43.4605	-3.7341	Somo, Ribamontan al Mar	Cantabria	MMA
16	8	2010	2	43.4035	-4.6545	Vidiago, Llanes	Asturias	MMA
17	8	2010	1	43.5583	-7.1706	Arealonga, Barreiros	Lugo	MMA
17	8	2010	1	43.4617	-4.9355	Cuevas del Mar, Llanes	Asturias	MMA
17	8	2010	4	43.3293	-1.9943	Donostia - San Sebastian	Guipuzcoa	MMA
17	8	2010	1	43.4703	-3.7742	El Camello, Santander	Cantabria	MMA
17	8	2010	1	43.4744	-3.7794	El Sardinero 1, Santander	Cantabria	MMA
17	8	2010	Nan	43.4792	-3.7826	El Sardinero 2	Cantabria	MMA
17	8	2010	4	43.4782	-3.6893	Langre, Ribamontan al Mar	Cantabria	MMA
17	8	2010	Nan	43.4782	-3.6893	Langre, Ribamontan al Mar	Cantabria	MMA
17	8	2010	2	43.4651	-3.7264	Loredo, Ribamontan al Mar	Cantabria	MMA
17	8	2010	Nan	43.4667	-3.7690	Los Bikinis, Santander	Cantabria	MMA
17	8	2010	1	43.6185	-5.7854	Luanco, Gozón	Asturias	MMA
17	8	2010	1	43.6185	-5.7854	Luanco, Gozón	Asturias	MMA
17	8	2010	Nan	43.3654	-3.1925	Mioño / Dícido	Cantabria	MMA
17	8	2010	1	43.4423	-4.8372	Niembro / Toranda, Llanes	Asturias	MMA
17	8	2010	1	43.4075	-3.3194	Oriñón, Castro Urdiales	Cantabria	MMA
17	8	2010	1	43.3974	-4.3282	Oyambre, Valdaliga	Cantabria	MMA
17	8	2010	2	43.4749	-3.7824	Piquio, Santander	Cantabria	MMA
17	8	2010	1	43.4605	-3.7341	Somo, Ribamontan al Mar	Cantabria	MMA
17	8	2010	1	43.4169	-3.3317	Sonabia, Castro Urdiales	Cantabria	MMA
17	8	2010	1	43.6276	-5.8793	Tenrero / Verdicio, Gozón	Asturias	MMA
17	8	2010	2	43.4571	-3.9730	Valdearenas, Piélagos	Cantabria	MMA
17	8	2010	2	43.4777	-3.8753	Virgen del Mar, Santander	Cantabria	MMA
18	8	2010	2	43.4378	-4.8248	Barro, Llanes	Asturias	MMA
18	8	2010	2	43.4368	-4.8099	Borizú, Llanes	Asturias	MMA
18	8	2010	1	43.4617	-4.9355	Cuevas del Mar, Llanes	Asturias	MMA
18	8	2010	4	43.3293	-1.9943	Donostia - San Sebastian	Guipuzcoa	MMA
18	8	2010	1	43.4601	-3.7591	Ribamontan al Mar	Cantabria	MMA
18	8	2010	1	43.4223	-4.7495	El Sablón, Llanes	Asturias	MMA
18	8	2010	1	43.4792	-3.7826	El Sardinero 2	Cantabria	MMA
18	8	2010	Nan	43.4744	-3.7794	El Sardinero, Santander	Cantabria	MMA
18	8	2010	2	43.5477	-5.5294	España, Villaviciosa	Asturias	MMA
18	8	2010	Nan	43.4821	-3.6782	Ribamontan al Mar	Cantabria	MMA
18	8	2010	2	43.4726	-3.7787	La Concha, Santander	Cantabria	MMA
18	8	2010	2	43.4234	-3.4355	La Salve, Laredo	Cantabria	MMA
18	8	2010	1	43.3965	-4.2206	Luaña / Cobreces	Cantabria	MMA
18	8	2010	3	43.4075	-3.3194	Oriñón, Castro Urdiales	Cantabria	MMA
18	8	2010	1	43.4340	-4.8067	Palombina, Llanes	Asturias	MMA
18	8	2010	1	43.5836	-5.9627	Salinas, Castrillón	Asturias	MMA
18	8	2010	1	43.5901	-5.9497	San Juan / Espartal	Asturias	MMA
18	8	2010	Nan	43.4605	-3.7341	Somo, Ribamontan al Mar	Cantabria	MMA
18	8	2010	3	43.4169	-3.3317	Sonabia, Castro Urdiales	Cantabria	MMA
18	8	2010	1	43.4280	-4.0950	Tagle / El Sable, Suances	Cantabria	MMA
18	8	2010	2	43.6085	-5.9294	Xago, Gozón	Asturias	MMA
19	8	2010	2	43.3752	-3.2107	Brazomar, Castro Urdiales	Cantabria	MMA
19	8	2010	4	43.3293	-1.9943	Donostia - San Sebastian	Guipuzcoa	MMA
19	8	2010	4	43.5084	-5.2595	La Griega, Colunga	Asturias	MMA
19	8	2010	2	43.5027	-5.2354	La Isla - Lastres, Colunga	Asturias	MMA
19	8	2010	2	43.4813	-5.2237	La Isla, Colunga	Asturias	MMA
19	8	2010	3	43.5096	-5.2684	Lastres, Colunga	Asturias	MMA
19	8	2010	4	43.4391	-4.0484	Los Locos, Suances	Cantabria	MMA
19	8	2010	2	43.3654	-3.1925	Mioño / Dicido	Cantabria	MMA
19	8	2010	3	43.3873	-3.2240	Ostende / Urdiales	Cantabria	MMA
19	8	2010	1	43.3854	-3.2142	Peñón de Santa Ana	Cantabria	MMA
19	8	2010	1	43.5634	-5.6858	Puerto de Gijón, Gijón	Asturias	MMA
19	8	2010	1	43.5767	-5.9961	Santa María del Mar	Asturias	MMA
19	8	2010	4	43.4680	-5.0707	Santa Marina, Ribadesella	Asturias	MMA
19	8	2010	1	43.4577	-3.8041	Santander	Cantabria	MMA
19	8	2010	2	43.4280	-4.0950	Tagle / El Sable, Suances	Cantabria	MMA
19	8	2010	2	43.4178	-4.7409	Toró, Llanes	Asturias	MMA
20	8	2010	1	43.3752	-3.2107	Brazomar, Castro Urdiales	Cantabria	MMA
20	8	2010	4	43.3293	-1.9943	Donostia - San Sebastian	Guipuzcoa	MMA
20	8	2010	1	43.4726	-3.7787	La Concha, Santander	Cantabria	MMA
20	8	2010	4	43.5084	-5.2595	La Griega, Colunga	Asturias	MMA
20	8	2010	1	43.4234	-3.4355	La Salve, Laredo	Cantabria	MMA
20	8	2010	1	43.5096	-5.2684	Lastres, Colunga	Asturias	MMA
20	8	2010	1	43.4864	-3.7870	Mataleñas, Santander	Cantabria	MMA
20	8	2010	2	43.3654	-3.1925	Mioño / Dicido	Cantabria	MMA
20	8	2010	3	43.4835	-3.7843	Molinucos, Santander	Cantabria	MMA
20	8	2010	1	43.4769	-5.1775	Morris, Caravia	Asturias	MMA
20	8	2010	Nan	43.3515	-3.1181	Muskiz / La Arena, Muskiz	Vizcaya	MMA
20	8	2010	2	43.3873	-3.2240	Ostende / Urdiales	Cantabria	MMA
20	8	2010	2	43.3974	-4.3282	Oyambre, Valdaliga	Cantabria	MMA
20	8	2010	4	43.4680	-5.0707	Santa Marina, Ribadesella	Asturias	MMA
20	8	2010	1	43.4744	-3.7794	Sardinero 1, Santander	Cantabria	MMA
20	8	2010	Nan	43.4169	-3.3317	Sonabia, Castro Urdiales	Cantabria	MMA
21	8	2010	1	43.4748	-3.9140	Arnia, Piélagos	Cantabria	MMA
21	8	2010	3	43.3812	-3.2138	Bahía, Castro Urdiales	Cantabria	MMA
21	8	2010	2	43.4368	-4.8099	Borizú, Llanes	Asturias	MMA
21	8	2010	3	43.3784	-3.2093	Castro Urdiales	Cantabria	MMA
21	8	2010	4	43.3293	-1.9943	Donostia - San Sebastian	Guipuzcoa	MMA
21	8	2010	Nan	43.4703	-3.7742	El Camello, Santander	Cantabria	MMA
21	8	2010	1	43.4601	-3.7591	Ribamontan al Mar	Cantabria	MMA
21	8	2010	Nan	43.4792	-3.7826	El Sardinero 2	Cantabria	MMA
21	8	2010	1	43.4768	-3.4768	Helguera, Noja	Cantabria	MMA
21	8	2010	1	43.4813	-5.2237	La Isla, Colunga	Asturias	MMA
21	8	2010	3	43.4234	-3.4355	La Salve, Laredo	Cantabria	MMA
21	8	2010	Nan	43.5049	-3.5449	Los Franceses, Arnuero	Cantabria	MMA
21	8	2010	Nan	43.4667	-3.7740	Magdalena, Santander	Cantabria	MMA
21	8	2010	1	43.4864	-3.7870	Mataleñas, Santander	Cantabria	MMA
21	8	2010	Nan	43.3515	-3.1181	Muskiz / La Arena, Muskiz	Vizcaya	MMA
21	8	2010	1	43.4340	-4.8067	Palombina, Llanes	Asturias	MMA
21	8	2010	1	43.4749	-3.7824	Piquio, Santander	Cantabria	MMA
21	8	2010	4	43.4376	-3.4514	San Martín, Santoña	Cantabria	MMA
21	8	2010	2	43.4605	-3.7341	Somo, Ribamontan al Mar	Cantabria	MMA
21	8	2010	1	43.4455	-4.8522	Torimbia, Llanes	Asturias	MMA
22	8	2010	1	43.3812	-3.2138	Bahía, Castro Urdiales	Cantabria	MMA
22	8	2010	1	43.4740	-3.9104	Covachos, Bezana	Cantabria	MMA
22	8	2010	4	43.3293	-1.9943	Donostia - San Sebastian	Guipuzcoa	MMA
22	8	2010	2	43.4015	-4.3573	El Cabo / Gerra	Cantabria	MMA
22	8	2010	2	43.4703	-3.7742	El Camello, Santander	Cantabria	MMA
22	8	2010	2	43.3864	-4.3889	El Rosal	Cantabria	MMA
22	8	2010	1	43.4744	-3.7794	El Sardinero 1, Santander	Cantabria	MMA
22	8	2010	1	43.4792	-3.7826	El Sardinero 2	Cantabria	MMA
22	8	2010	Nan	43.4726	-3.7787	La Concha, Santander	Cantabria	MMA

22	8	2010	1	43.4749	-3.7824	Piquio, Santander	Cantabria	MMA
22	8	2010	Nan	43.4280	-4.0950	Tagle / El Sable, Suances	Cantabria	MMA
22	8	2010	1	43.4377	-4.8186	Troenzo, Llanes	Asturias	MMA
22	8	2010	1	43.4777	-3.8753	Virgen del Mar, Santander	Cantabria	MMA
23	8	2010	Nan	43.3914	-4.2862	Comillas, Comillas	Cantabria	MMA
23	8	2010	2	43.3654	-3.1925	Dicido, Castro Urdiales	Cantabria	MMA
23	8	2010	4	43.3293	-1.9943	Donostia - San Sebastian	Guipuzcoa	MMA
23	8	2010	3	43.4601	-3.7591	Ribamontan al Mar	Cantabria	MMA
23	8	2010	2	43.4234	-3.4355	La Salve, Laredo	Cantabria	MMA
23	8	2010	1	43.4782	-3.6893	Ribamontan al Mar	Cantabria	MMA
23	8	2010	1	43.4667	-3.7690	Los Bikinis, Santander	Cantabria	MMA
23	8	2010	3	43.4864	-3.7870	Mataleñas, Santander	Cantabria	MMA
23	8	2010	1	43.3873	-3.2240	Ostende / Urdiales	Cantabria	MMA
23	8	2010	2	43.3974	-4.3282	Oyambre, Valdaliga	Cantabria	MMA
23	8	2010	2	43.3504	-3.0507	Puerto Santurtzi, Santurtzi	Vizcaya	MMA
23	8	2010	2	43.4605	-3.7341	Somo, Ribamontan al Mar	Cantabria	MMA
23	8	2010	1	43.4777	-3.8753	Virgen del Mar, Santander	Cantabria	MMA
24	8	2010	2	43.4605	-3.7991	Cabo Mayor, Santander	Cantabria	MMA
24	8	2010	5	43.3293	-1.9943	Donostia - San Sebastian	Guipuzcoa	MMA
24	8	2010	2	43.4703	-3.7742	El Camello, Santander	Cantabria	MMA
24	8	2010	2	43.4782	-3.6893	Langre 1	Cantabria	MMA
24	8	2010	1	43.4749	-3.7824	Piquio, Santander	Cantabria	MMA
25	8	2010	2	43.4039	-3.3133	Arenillas / Islares	Cantabria	MMA
25	8	2010	1	43.3752	-3.2107	Brazomar, Castro Urdiales	Cantabria	MMA
25	8	2010	1	43.3654	-3.1925	Dicido, Castro Urdiales	Cantabria	MMA
25	8	2010	4	43.3293	-1.9943	Donostia - San Sebastian	Guipuzcoa	MMA
25	8	2010	2	43.4703	-3.7742	El Camello, Santander	Cantabria	MMA
25	8	2010	2	43.4601	-3.7591	Ribamontan al Mar	Cantabria	MMA
25	8	2010	2	43.4726	-3.7787	La Concha, Santander	Cantabria	MMA
25	8	2010	1	43.4372	-4.0410	La Concha, Suances	Cantabria	MMA
25	8	2010	2	43.4320	-4.0341	La Ribera, Suances	Cantabria	MMA
25	8	2010	2	43.4234	-3.4355	La Salve, Laredo	Cantabria	MMA
25	8	2010	2	43.4782	-3.6893	Ribamontan al Mar	Cantabria	MMA
25	8	2010	1	43.6185	-5.7854	Luanco, Gozón	Asturias	MMA
25	8	2010	2	43.3965	-4.2206	Luaña / Cobreces	Cantabria	MMA
25	8	2010	4	43.4864	-3.7870	Mataleñas, Santander	Cantabria	MMA
25	8	2010	1	43.4835	-3.7843	Molinucos, Santander	Cantabria	MMA
25	8	2010	1	43.3873	-3.2240	Ostende / Urdiales	Cantabria	MMA
25	8	2010	2	43.4749	-3.7824	Piquio, Santander	Cantabria	MMA
25	8	2010	2	43.4744	-3.7794	Sardinero 1, Santander	Cantabria	MMA
25	8	2010	2	43.4792	-3.7826	Sardinero 2, Santander	Cantabria	MMA
26	8	2010	Nan	43.4573	-3.9623	Canallave, Pielagos	Cantabria	MMA
26	8	2010	4	43.3293	-1.9943	Donostia - San Sebastian	Guipuzcoa	MMA
26	8	2010	2	43.4605	-3.7991	Embarcadero, Santander	Cantabria	MMA
26	8	2010	3	43.4605	-3.7991	Ferri-Nautico Pesquero	Cantabria	MMA
26	8	2010	2	43.4372	-4.0410	La Concha, Suances	Cantabria	MMA
26	8	2010	1	43.5084	-5.2595	La Griega, Colunga	Asturias	MMA
26	8	2010	2	43.4234	-3.4355	La Salve, Laredo	Cantabria	MMA
26	8	2010	1	43.5096	-5.2684	Lastres, Colunga	Asturias	MMA
26	8	2010	2	43.4641	-3.7781	Los Peligros, Santander	Cantabria	MMA
26	8	2010	1	43.4667	-3.7740	Magdalena, Santander	Cantabria	MMA
26	8	2010	1	43.4075	-3.3194	Oriñón, Castro Urdiales	Cantabria	MMA
26	8	2010	2	43.4169	-3.3317	Sonabia, Castro Urdiales	Cantabria	MMA
26	8	2010	Nan	43.4571	-3.9730	Valdearenas, Piélagos	Cantabria	MMA
27	8	2010	Nan	43.3566	-3.0219	Arrigunaga, Getxo	Vizcaya	MMA
27	8	2010	1	43.3654	-3.1925	Dicido, Castro Urdiales	Cantabria	MMA
27	8	2010	4	43.3293	-1.9943	Donostia - San Sebastian	Guipuzcoa	MMA
27	8	2010	2	43.4667	-3.7690	Los Bikinis, Santander	Cantabria	MMA
27	8	2010	Nan	43.3941	-4.3803	San Vicente de la Barquera	Cantabria	MMA
27	8	2010	2	43.4460	-4.8702	San Antolín, Llanes	Asturias	MMA
27	8	2010	3	43.4338	-4.7889	San Martín, Llanes	Asturias	MMA
27	8	2010	1	43.4571	-3.9730	Valdearenas, Piélagos	Cantabria	MMA
27	8	2010	3	43.3994	-4.3668	San Vicente de la Barquera	Cantabria	MMA
28	8	2010	2	43.4748	-3.9140	Arnia, Piélagos	Cantabria	MMA
28	8	2010	3	43.4577	-3.8041	Bahía de Santander	Cantabria	MMA
28	8	2010	2	43.4792	-3.7826	Castañeda, Santander	Cantabria	MMA
28	8	2010	1	43.3914	-4.2862	Comillas, Comillas	Cantabria	MMA
28	8	2010	Nan	43.4740	-3.9104	Covachos, Bezana	Cantabria	MMA
28	8	2010	4	43.5010	-3.6130	Cuberris, Bareyo	Cantabria	MMA
28	8	2010	4	43.3293	-1.9943	Donostia - San Sebastian	Guipuzcoa	MMA
28	8	2010	2	43.4703	-3.7742	El Camello, Santander	Cantabria	MMA
28	8	2010	3	43.4601	-3.7591	El Puntal	Cantabria	MMA
28	8	2010	2	43.4744	-3.7794	El Sardinero 1, Santander	Cantabria	MMA
28	8	2010	2	43.4821	-3.6782	La Canal	Cantabria	MMA
28	8	2010	3	43.4726	-3.7787	La Concha, Santander	Cantabria	MMA
28	8	2010	3	43.4234	-3.4355	La Salve, Laredo	Cantabria	MMA
28	8	2010	2	43.4782	-3.6893	Langre, Ribamontan al Mar	Cantabria	MMA
28	8	2010	2	43.4651	-3.7264	Loredo, Ribamontan al Mar	Cantabria	MMA
28	8	2010	Nan	43.4667	-3.7740	Magdalena, Santander	Cantabria	MMA
28	8	2010	2	43.4864	-3.7870	Mataleñas, Santander	Cantabria	MMA
28	8	2010	3	43.3974	-4.3282	Oyambre, Valdaliga	Cantabria	MMA
28	8	2010	2	43.4749	-3.7824	Piquio, Santander	Cantabria	MMA
28	8	2010	2	43.4745	-3.8910	San Juan de la Canal	Cantabria	MMA
28	8	2010	1	43.4605	-3.7341	Somo, Ribamontan al Mar	Cantabria	MMA
28	8	2010	2	43.4571	-3.9730	Valdearenas, Piélagos	Cantabria	MMA
28	8	2010	2	43.4777	-3.8753	Virgen del Mar, Santander	Cantabria	MMA
29	8	2010	2	43.4748	-3.9140	Arnia, Piélagos	Cantabria	MMA
29	8	2010	2	43.4368	-4.8099	Borizú, Llanes	Asturias	MMA
29	8	2010	2	43.4605	-3.7991	Cabo Mayor, Santander	Cantabria	MMA
29	8	2010	2	43.3914	-4.2862	Comillas, Comillas	Cantabria	MMA
29	8	2010	1	43.4740	-3.9104	Covachos, Bezana	Cantabria	MMA
29	8	2010	2	43.3654	-3.1925	Dicido, Castro Urdiales	Cantabria	MMA
29	8	2010	4	43.3293	-1.9943	Donostia - San Sebastian	Guipuzcoa	MMA
29	8	2010	2	43.4703	-3.7742	El Camello, Santander	Cantabria	MMA
29	8	2010	2	43.4601	-3.7591	El Puntal	Cantabria	MMA
29	8	2010	3	43.5074	-3.5786	La Arena, Arnuero	Cantabria	MMA
29	8	2010	1	43.4372	-4.0410	La Concha, Suances	Cantabria	MMA
29	8	2010	1	43.5084	-5.2595	La Griega, Colunga	Asturias	MMA
29	8	2010	1	43.4320	-4.0341	La Ribera, Suances	Cantabria	MMA
29	8	2010	1	43.4782	-3.6893	Langre, Ribamontan al Mar	Cantabria	MMA
29	8	2010	Nan	43.4667	-3.7690	Los Bikinis, Santander	Cantabria	MMA
29	8	2010	1	43.4749	-3.7824	Piquio, Santander	Cantabria	MMA
29	8	2010	1	43.4332	-4.7854	Poó, Llanes	Asturias	MMA
29	8	2010	2	43.4195	-4.7471	Puertu Chicu, Llanes	Asturias	MMA
29	8	2010	3	43.4605	-3.7341	Somo, Ribamontan al Mar	Cantabria	MMA
29	8	2010	1	43.4178	-4.7409	Toró, Llanes	Asturias	MMA
29	8	2010	2	43.4777	-3.8753	Virgen del Mar, Santander	Cantabria	MMA
30	8	2010	1	43.4137	-4.7114	Ballota, Llanes	Asturias	MMA
30	8	2010	2	43.4378	-4.8248	Barro, Llanes	Asturias	MMA
30	8	2010	3	43.4368	-4.8099	Borizú, Llanes	Asturias	MMA
30	8	2010	1	43.4573	-3.9623	Canallave, Pielagos	Cantabria	MMA
30	8	2010	1	43.5788	-5.7426	Carranques, Carreño	Asturias	MMA
30	8	2010	1	43.4223	-4.7495	El Sablón, Llanes	Asturias	MMA
30	8	2010	1	43.4744	-3.7794	El Sardinero, Santander	Cantabria	MMA
30	8	2010	2	43.4234	-3.4355	La Salve, Laredo	Cantabria	MMA
30	8	2010	2	43.5096	-5.2684	Lastres, Colunga	Asturias	MMA
30	8	2010	2	43.4075	-3.3194	Oriñón, Castro Urdiales	Cantabria	MMA
30	8	2010	1	43.3873	-3.2240	Ostende, Castro Urdiales	Cantabria	MMA

30	8	2010	3	43.3974	-4.3282	Oyambre, Valdaliga	Cantabria	MMA
30	8	2010	2	43.4332	-4.7854	Poó, Llanes	Asturias	MMA
30	8	2010	2	43.4601	-4.9264	San Antonio, Llanes	Asturias	MMA
30	8	2010	4	43.4338	-4.7889	San Martín, Llanes	Asturias	MMA
30	8	2010	3	43.4423	-4.8372	Toranda / Niembro, Llanes	Asturias	MMA
31	8	2010	2	43.4132	-4.7067	Andrín, Llanes	Asturias	MMA
31	8	2010	2	43.3752	-3.2107	Brazomar, Castro Urdiales	Cantabria	MMA
31	8	2010	2	43.3831	-3.2114	El Rompeolas	Cantabria	MMA
31	8	2010	1	43.4223	-4.7495	El Sablón, Llanes	Asturias	MMA
31	8	2010	1	43.4726	-3.7787	La Concha, Santander	Cantabria	MMA
31	8	2010	5	43.4234	-3.4355	La Salve, Laredo	Cantabria	MMA
31	8	2010	1	43.3965	-4.2206	Luaña, Alfoz de Lloredo	Cantabria	MMA
31	8	2010	Nan	43.4667	-3.7740	Magdalena, Santander	Cantabria	MMA
31	8	2010	1	43.4864	-3.7870	Mataleñas, Santander	Cantabria	MMA
31	8	2010	1	43.3873	-3.2240	Ostende, Castro Urdiales	Cantabria	MMA
31	8	2010	3	43.3974	-4.3282	Oyambre, Valdaliga	Cantabria	MMA
2	9	2010	1	43.3752	-3.2107	Brazomar, Castro Urdiales	Cantabria	MMA
2	9	2010	1	43.4821	-3.6782	Galizano	Cantabria	MMA
2	9	2010	2	43.4234	-3.4355	La Salve, Laredo	Cantabria	MMA
2	9	2010	1	43.3873	-3.2240	Ostende, Castro Urdiales	Cantabria	MMA
3	9	2010	2	43.4234	-3.4355	La Salve, Laredo	Cantabria	MMA
4	9	2010	2	43.5010	-3.6130	Cuberris, Bareyo	Cantabria	MMA
4	9	2010	1	43.4234	-3.4355	Laredo, Laredo	Cantabria	MMA
4	9	2010	1	43.4340	-4.8067	Palombina, Llanes	Asturias	MMA
5	9	2010	2	43.3654	-3.1925	Dicido, Castro Urdiales	Cantabria	MMA
5	9	2010	1	43.4234	-3.4355	La Salve, Laredo	Cantabria	MMA
6	9	2010	1	43.4821	-3.6782	Galizano	Asturias	MMA
6	9	2010	2	43.4234	-3.4355	La Salve, Laredo	Asturias	MMA
8	9	2010	2	43.4605	-3.7991	Cabo Mayor, Santander	Cantabria	MMA
8	9	2010	1	43.5901	-5.9497	San Juan / Espartal	Asturias	MMA
9	9	2010	2	43.4782	-3.6893	Langre, Ribamontan al Mar	Asturias	MMA
10	9	2010	2	43.4792	-3.7826	Castañeda, Santander	Asturias	MMA
10	9	2010	1	43.4864	-3.7870	Mataleñas, Santander	Asturias	MMA
10	9	2010	Nan	43.4749	-3.7824	Piquio, Santander	Asturias	MMA
10	9	2010	1	43.4744	-3.7794	Sardinero 1, Santander	Asturias	MMA
11	9	2010	1	43.4864	-3.7870	Mataleñas, Santander	Asturias	MMA
11	9	2010	2	43.4835	-3.7843	Molinucos, Santander	Asturias	MMA
13	9	2010	2	43.4726	-3.7787	La Concha, Santander	Cantabria	MMA
13	9	2010	2	43.4234	-3.4355	La Salve, Laredo	Cantabria	MMA
13	9	2010	2	43.4749	-3.7824	Piquio, Santander	Cantabria	MMA
13	9	2010	2	43.4744	-3.7794	Sardinero 1, Santander	Cantabria	MMA
13	9	2010	2	43.4792	-3.7826	Sardinero 2, Santander	Cantabria	MMA
14	9	2010	2	43.4234	-3.4355	La Salve, Laredo	Cantabria	MMA
14	9	2010	1	43.3854	-3.2142	Peñón de Santa Ana	Cantabria	MMA
15	9	2010	2	43.4039	-2.6355	Elantxobe	Vizcaya	MMA
15	9	2010	Nan	43.4110	-2.6584	Laga, Ibarrangelu	Vizcaya	MMA
15	9	2010	1	28.3786	-16.7273	San Marcos	Tenerife	MMA
20	9	2010	Nan	43.3643	-2.4930	Karraspio, Mendexa	Vizcaya	MMA
22	9	2010	3	43.5452	-5.6538	Gijón	Asturias	MMA
22	9	2010	2	43.6325	-5.7798	Punta de la Vaca, Gozón	Asturias	MMA
26	9	2010	2	43.4713	-3.4631	Berria, Santoña	Cantabria	MMA
26	9	2010	2	43.5074	-3.5786	La Arena, Arnuero	Cantabria	MMA
8	2	2011	1	36.7376	-3.8044	City	Málaga	Consejería de Medio Ambiente
8	2	2011	1	36.7055	-3.8219	City	Málaga	Consejería de Medio Ambiente
8	3	2011	5	28.4129	-16.5616	Jardín, Puerto de la Cruz	St Cruz Tenerife	http://www.rtvc.es/noticias/invasi%C3%B3n-de-medudas-en-la-paya-jard%C3%ADn-de-puerto-de-la-cruz-66165.aspx
18	3	2011	4	28.5077	-16.1834	Las Teresitas	St Cruz Tenerife	http://www.laopinion.es/tenerife/2011/03/22/desaconsejan-bano-llegada-aguavivas-playa/335663.html
19	3	2011	4	28.5077	-16.1834	Las Teresitas	St Cruz Tenerife	http://www.rtvc.es/noticias/invasi%C3%B3n-de-medusas-66772.aspx
19	3	2011	4	28.1422	-15.4386	Las Canteras	Gran Canaria	http://www.rtvc.es/noticias/invasi%C3%B3n-de-medusas-66772.aspx
20	3	2011	4	28.5077	-16.1834	Las Teresitas	St Cruz Tenerife	http://www.laopinion.es/tenerife/2011/03/22/desaconsejan-bano-llegada-aguavivas-playa/335663.html
29	3	2011	4	28.2506	-16.3818	Punta Prieta (Güimar)	St Cruz Tenerife	http://www.eldia.es/2011-03-30/ULTIMA/8-Invasion-medusas.htm
29	3	2011	4	28.2446	-16.3948	Chimaje (Güimar)	St Cruz Tenerife	http://www.eldia.es/2011-03-30/ULTIMA/8-Invasion-medusas.htm
29	3	2011	4	28.2404	-16.4010	Tablado (Güimar)	St Cruz Tenerife	http://www.eldia.es/2011-03-30/ULTIMA/8-Invasion-medusas.htm
2	4	2011	4	28.1422	-15.4386	Las Canteras	Gran Canaria	http://www.rtvc.es/noticias/m%C3%A1s-de-40-casos-de-picadura-de-medusas-en-las-canteras-67707.aspx
21	4	2011	1	36.7279	-3.7229	Marina del Este, Almuñecar	Granada	Aquatours
7	5	2011	1	38.5530	0.1978	North and center of the province	Alicante	http://www.lasprovincias.es/v/20110528/valencia/peligrosa-medusa-amenaza-nuevo-20110528.html
11	5	2011	1	38.5530	0.1978	North and center of the province	Alicante	http://www.lasprovincias.es/v/20110528/valencia/peligrosa-medusa-amenaza-nuevo-20110528.html
5	6	2011	1	38.4200	0.3475	El Campello	Alicante	Cudomar SL
16	6	2011	1	36.1619	-5.3590	Poniente (La línea)	Cádiz	http://www.europasur.es/article/lalinea/1000168/la/playa/poniente/es/cerrada/bano/por/medusas.html
1	7	2011	2	43.3177	-2.0011	Ondarreta	Guipúzcoa	http://www.diariovasco.com/20110705/local/heridos-leves-picaduras-medusas-201107051840.html
4	7	2011	2	43.3177	-2.0011	Ondarreta	Guipúzcoa	http://www.diariovasco.com/20110705/local/heridos-leves-picaduras-medusas-201107051840.html
5	7	2011	4	43.3151	-2.0506	Igueldo	Guipúzcoa	http://www.diariovasco.com/20110705/local/heridos-leves-picaduras-medusas-201107051840.html
7	7	2011	3	43.3287	-2.3887	Ondarroa	Vizcaya	http://www.adn.es/local/bilbao/20110708/NWS-0222-Alerta-mejillones-medusas-cebra.html
8	7	2011	4	43.4646	-3.7729	Magdalena	Cantabria	http://www.eldiariomontanes.es/v/20110709/cantabria/otras-noticias/carabela-portuguesa-llega-costa-20110709.html
9	7	2011	5	43.3875	-3.2224	Castro Urdiales	Cantabria	http://www.eldiariomontanes.es/v/20110710/cantabria/otras-noticias/carabelas-portuguesas-invaden-playas-20110710.html
12	7	2011	5	43.3177	-2.0011	Ondarreta	Guipúzcoa	http://www.20minutos.es/noticia/1108752/0/
14	7	2011	5	43.2878	-2.1707	Zarautz	Guipúzcoa	http://www.diariovasco.com/v/20110714/bajo-deba/carabela-portuguesa-llegado-playas-20110714.html
19	7	2011	5	43.3288	-1.9759	Zurriola	Guipúzcoa	http://www.diariovasco.com/v/20110720/al-dia-local/llegan-carabelas-sebastian-20110720.html
8	10	2011	3	28.1076	-15.7070	Las Salinas (Agaete)	Gran Canaria	http://www.infonortedigital.com/portada/component/content/article/6-interes/8605-advierten-del-peligro-de-medusas-en-la-zona-de-las-salinas-en-agaete
12	5	2012	4	41.5629	2.5201	Sant Vicenç de Montalt	Barcelona	http://www.lavanguardia.com/medio-ambiente/20120512/54293011130/medusas-playa-sant-vicenc-de-montalt.html
20	8	2012	5	43.5532	-6.3696	Cadavedo, Valdés	Asturias	http://www.abc.es/20120821/sociedad/abci-medusas-playas-asturias-galicia-201208211008.html
20	8	2012	5	43.5532	-6.3696	Cadavedo, Valdés	Asturias	http://www.abc.es/20120821/sociedad/abci-medusas-playas-asturias-galicia-201208211008.html
5	9	2012	5	43.5546	-7.1487	Esteiro, Ribadeo	Lugo	http://www.lavozdegalicia.es/noticia/amarina/2012/09/06/playas-ribadeo-barreiros-foz-burela-plagadas-medusas/0003_201209X6C7996.htm
5	9	2012	5	43.5566	-7.1368	As Illas, Ribadeo	Lugo	http://www.lavozdegalicia.es/noticia/amarina/2012/09/06/playas-ribadeo-barreiros-foz-burela-plagadas-medusas/0003_201209X6C7996.htm
5	9	2012	5	43.5295	-7.0417	Os Bloques, Ribadeo	Lugo	http://www.lavozdegalicia.es/noticia/amarina/2012/09/06/playas-ribadeo-barreiros-foz-burela-plagadas-medusas/0003_201209X6C7996.htm
5	9	2012	5	43.5559	-7.1342	Os Castro, Ribadeo	Lugo	http://www.lavozdegalicia.es/noticia/amarina/2012/09/06/playas-ribadeo-barreiros-foz-burela-plagadas-medusas/0003_201209X6C7996.htm
5	9	2012	5	43.5550	-7.1613	As Catedrais, Ribadeo	Lugo	http://www.lavozdegalicia.es/noticia/amarina/2012/09/06/playas-ribadeo-barreiros-foz-burela-plagadas-medusas/0003_201209X6C7996.htm
5	9	2012	5	43.5612	-7.1907	Barreiros	Lugo	http://www.lavozdegalicia.es/noticia/amarina/2012/09/06/playas-ribadeo-barreiros-foz-burela-plagadas-medusas/0003_201209X6C7996.htm
5	9	2012	5	43.5786	-7.2493	A Rapadoira, Foz	Lugo	http://www.lavozdegalicia.es/noticia/amarina/2012/09/06/playas-ribadeo-barreiros-foz-burela-plagadas-medusas/0003_201209X6C7996.htm
5	9	2012	5	43.5814	-7.2606	Llas, Foz	Lugo	http://www.lavozdegalicia.es/noticia/amarina/2012/09/06/playas-ribadeo-barreiros-foz-burela-plagadas-medusas/0003_201209X6C7996.htm
5	9	2012	5	43.5887	-7.2785	Peizas, Foz	Lugo	http://www.lavozdegalicia.es/noticia/amarina/2012/09/06/playas-ribadeo-barreiros-foz-burela-plagadas-medusas/0003_201209X6C7996.htm
5	9	2012	5	43.6735	-7.3629	Ril, Burela	Lugo	http://www.lavozdegalicia.es/noticia/amarina/2012/09/06/playas-ribadeo-barreiros-foz-burela-plagadas-medusas/0003_201209X6C7996.htm
5	9	2012	5	43.6640	-7.3560	Penaural, Burela	Lugo	http://www.lavozdegalicia.es/noticia/amarina/2012/09/06/playas-ribadeo-barreiros-foz-burela-plagadas-medusas/0003_201209X6C7996.htm
5	9	2012	5	43.6663	-7.3565	O Portelo, Burela	Lugo	http://www.lavozdegalicia.es/noticia/amarina/2012/09/06/playas-ribadeo-barreiros-foz-burela-plagadas-medusas/0003_201209X6C7996.htm
6	9	2012	5	43.5546	-7.1487	Esteiro, Ribadeo	Lugo	http://www.lavozdegalicia.es/noticia/galicia/2012/09/06/plaga-medusas-mantiene-cerradas-20-playas-marina/0003134696167995760 8212.htm
6	9	2012	5	43.5566	-7.1368	As Illas, Ribadeo	Lugo	http://www.lavozdegalicia.es/noticia/galicia/2012/09/06/plaga-medusas-mantiene-cerradas-20-playas-marina/00031346961679957608212.htm

6	9	2012	5	43.5295	-7.0417	Os Bloques, Ribadeo	Lugo	http://www.lavozdegalicia.es/noticia/galicia/2012/09/06/plaga-medusas-mantiene-cerradas-20-playas-marina/00031346961679957608212.htm
6	9	2012	5	43.5559	-7.1342	Os Castro, Ribadeo	Lugo	http://www.lavozdegalicia.es/noticia/galicia/2012/09/06/plaga-medusas-mantiene-cerradas-20-playas-marina/00031346961679957608212.htm
6	9	2012	5	43.5550	-7.1613	As Catedrais, Ribadeo	Lugo	http://www.lavozdegalicia.es/noticia/galicia/2012/09/06/plaga-medusas-mantiene-cerradas-20-playas-marina/00031346961679957608212.htm
6	9	2012	5	43.5612	-7.1907	Barreiros	Lugo	http://www.lavozdegalicia.es/noticia/galicia/2012/09/06/plaga-medusas-mantiene-cerradas-20-playas-marina/00031346961679957608212.htm
6	9	2012	5	43.5786	-7.2493	A Rapadoira, Foz	Lugo	http://www.lavozdegalicia.es/noticia/galicia/2012/09/06/plaga-medusas-mantiene-cerradas-20-playas-marina/00031346961679957608212.htm
6	9	2012	5	43.5814	-7.2606	Llas, Foz	Lugo	http://www.lavozdegalicia.es/noticia/galicia/2012/09/06/plaga-medusas-mantiene-cerradas-20-playas-marina/00031346961679957608212.htm
6	9	2012	5	43.5887	-7.2785	Peizas, Foz	Lugo	http://www.lavozdegalicia.es/noticia/galicia/2012/09/06/plaga-medusas-mantiene-cerradas-20-playas-marina/00031346961679957608212.htm
6	9	2012	5	43.6735	-7.3629	Ril, Burela	Lugo	http://www.lavozdegalicia.es/noticia/galicia/2012/09/06/plaga-medusas-mantiene-cerradas-20-playas-marina/00031346961679957608212.htm
6	9	2012	5	43.6640	-7.3560	Penaural, Burela	Lugo	http://www.lavozdegalicia.es/noticia/galicia/2012/09/06/plaga-medusas-mantiene-cerradas-20-playas-marina/00031346961679957608212.htm
6	9	2012	5	43.6663	-7.3565	O Portelo, Burela	Lugo	http://www.lavozdegalicia.es/noticia/galicia/2012/09/06/plaga-medusas-mantiene-cerradas-20-playas-marina/00031346961679957608212.htm
7	9	2012	5	43.5546	-7.1487	Esteiro, Ribadeo	Lugo	http://www.abc.es/agencias/noticia.asp?noticia=1245907
7	9	2012	5	43.5566	-7.1368	As Illas, Ribadeo	Lugo	http://www.abc.es/agencias/noticia.asp?noticia=1245908
7	9	2012	5	43.5295	-7.0417	Os Bloques, Ribadeo	Lugo	http://www.abc.es/agencias/noticia.asp?noticia=1245909
7	9	2012	5	43.5559	-7.1342	Os Castro, Ribadeo	Lugo	http://www.abc.es/agencias/noticia.asp?noticia=1245910
7	9	2012	5	43.5550	-7.1613	As Catedrais, Ribadeo	Lugo	http://www.abc.es/agencias/noticia.asp?noticia=1245911
7	9	2012	5	43.5612	-7.1907	Barreiros	Lugo	http://www.abc.es/agencias/noticia.asp?noticia=1245912
7	9	2012	5	43.5786	-7.2493	A Rapadoira, Foz	Lugo	http://www.abc.es/agencias/noticia.asp?noticia=1245913
7	9	2012	5	43.5814	-7.2606	Llas, Foz	Lugo	http://www.abc.es/agencias/noticia.asp?noticia=1245914
7	9	2012	5	43.5887	-7.2785	Peizas, Foz	Lugo	http://www.abc.es/agencias/noticia.asp?noticia=1245915
7	9	2012	5	43.6735	-7.3629	Ril, Burela	Lugo	http://www.abc.es/agencias/noticia.asp?noticia=1245916
7	9	2012	5	43.6640	-7.3560	Penaural, Burela	Lugo	http://www.abc.es/agencias/noticia.asp?noticia=1245917
7	9	2012	5	43.6663	-7.3565	O Portelo, Burela	Lugo	http://www.abc.es/agencias/noticia.asp?noticia=1245918
26	9	2012	5	43.5451	-5.6531	San Lorenzo, Gijón	Asturias	http://www.elcomercio.es/v/20120927/gijon/plaga-medusas-obliga-cerrar-20120927.html
29	9	2012	5	43.5451	-5.6531	San Lorenzo, Gijón	Asturias	http://www.elcomercio.es/v/20120930/gijon/medusas-cierran-playas-20120930.html
29	9	2012	5	43.5455	-5.6925	Arbeyal, Gijón	Asturias	http://www.elcomercio.es/v/20120930/gijon/medusas-cierran-playas-20120930.html
29	9	2012	5	43.5432	-5.6709	Poniente, Gijón	Asturias	http://www.elcomercio.es/v/20120930/gijon/medusas-cierran-playas-20120930.html
29	9	2012	5	43.5874	-5.7594	Candás	Asturias	http://www.elcomercio.es/v/20120930/gijon/medusas-cierran-playas-20120930.html

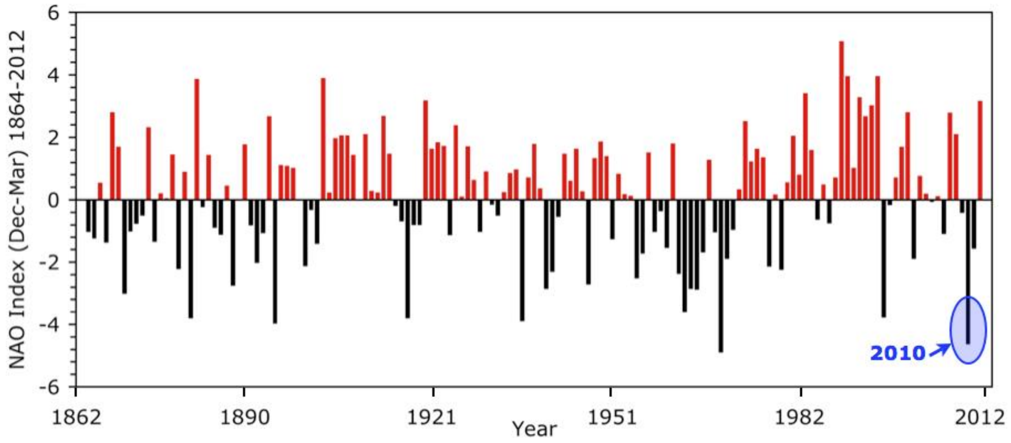

Supplementary Figure 1 | Winter North Atlantic Oscillation index. Winter index of NAO based on the difference of the normalized sea level pressure (SLP) between Lisbon, Portugal and Stykkisholmur/Reykjavik, Iceland, since 1864. The station index value for year N refers to an average of December of year N-1 and January, February and March year of N. The SLP anomalies at each station were normalized by the division of each seasonal mean pressure by the long-term mean (1864-1983) standard deviation. Normalization is used to avoid the series being dominated by the greater variability of the northern station. (From Hurrel, 2012[6]).

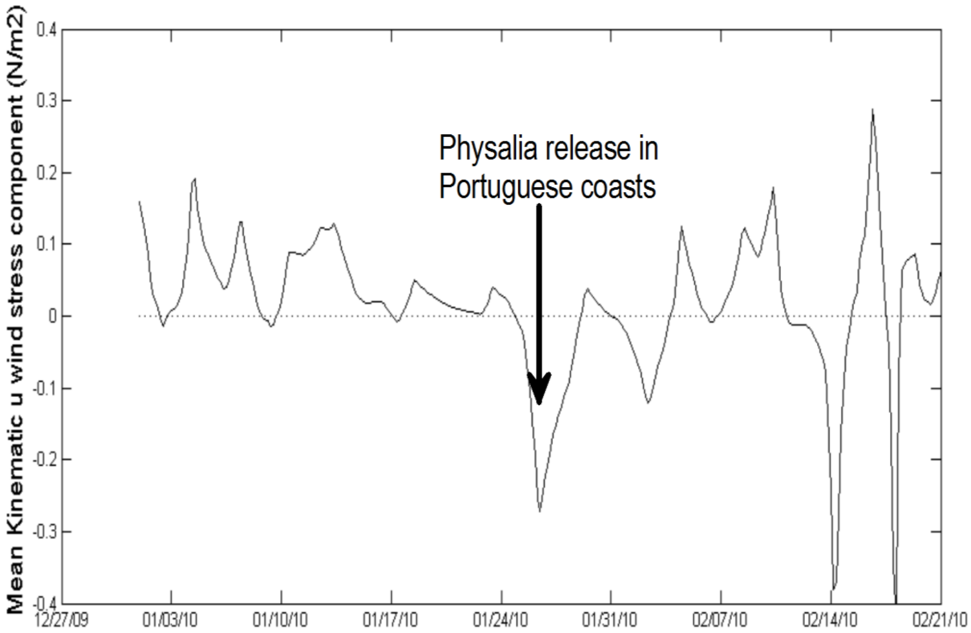

Supplementary Figure 2 | Wind pattern along the southwest Portugal shore. Daily mean kinematic wind stress (u component) at -10°W between 37° and 38°N one month before the main stranding event of *P. physalis* at the Doñana National Park shore. Westerlies are denoted by positive values and easterlies by negative values.

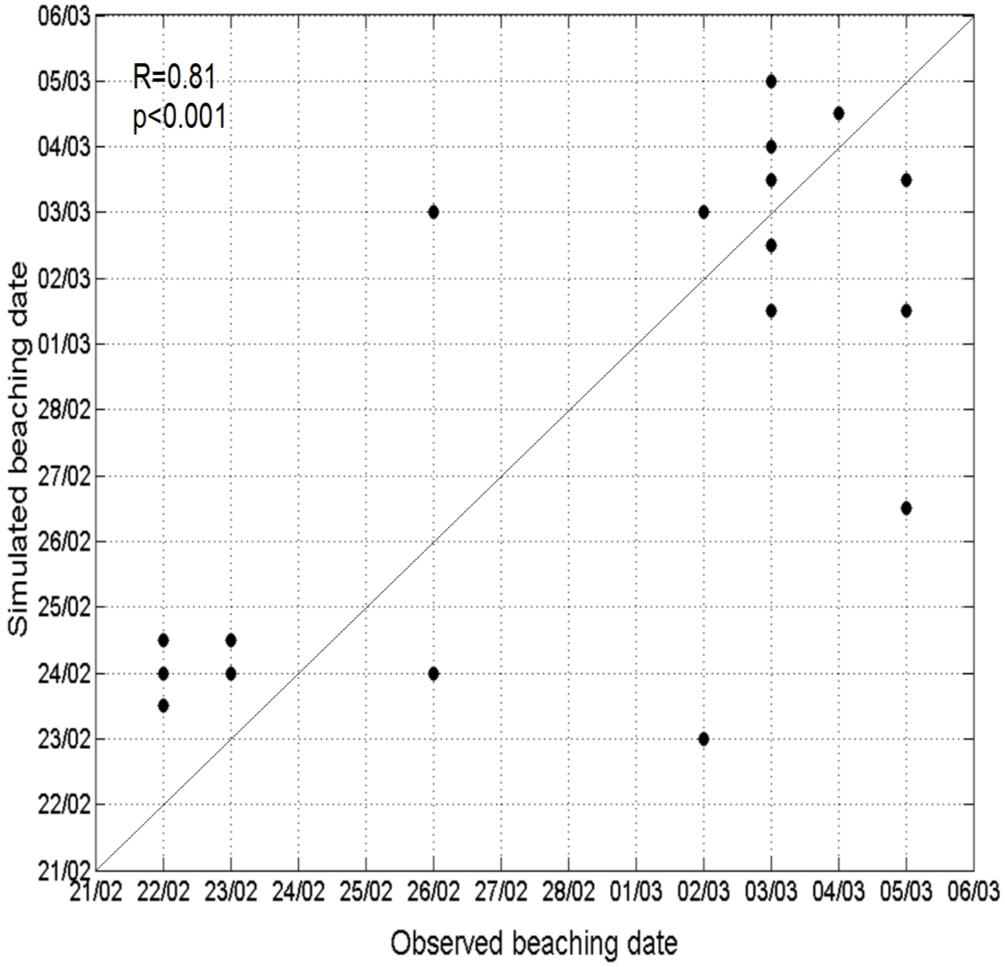

Supplementary Figure 3 | Comparison between observed and simulated beaching data of Portuguese Man-of-War. The oceanic population of *P. physalis* started to appear as stranded on the beach on 22 February 2010. The observations on the coast occurred from west to east, advancing toward the Mediterranean, passing through the Strait of Gibraltar and to the easternmost Alboran Sea. The observed timing of beaching is highly correlated to the simulated timing ($r=0.81$, $p<0.001$, $n= 18$).
